# Immunomodulatory Properties of Blackberry Anthocyanins in THP-1 Derived Macrophages

**DOI:** 10.3390/ijms221910483

**Published:** 2021-09-28

**Authors:** Ebru Cenk, Cornelia Schmutz, Gudrun Pahlke, Anne Oertel, Jessica Kollarova, Hans-Peter Mock, Andrea Matros, Doris Marko

**Affiliations:** 1 Department of Food Chemistry and Toxicology, Faculty of Chemistry, University of Vienna, 1090 Vienna, Austria; ebru.cenk@univie.ac.at (E.C.); cornelia.schmutz@univie.ac.at (C.S.); gudrun.pahlke@univie.ac.at (G.P.); kollarova.jessica@yahoo.de (J.K.); 2 Department of Physiology and Cell Biology, Leibniz Institute of Plant Genetics and Crop Plant Research (IPK-Gatersleben), Corrensstr. 3, D-06466 Gatersleben, Germany; oertel@burg-halle.de (A.O.); mock@ipk-gatersleben.de (H.-P.M.); andrea.matros@adelaide.edu.au (A.M.); 3 BioLab Platform for Living Matter, University of Art and Design, Neuwerk 7, D-06108 Halle (Saale), Germany; 4 School of Agriculture, Food and Wine, Waite Campus, University of Adelaide, Adelaide, SA 5064, Australia

**Keywords:** anthocyanin, blackberry, cytokine, microRNA, NF-κB, LC-MS, inflammation, polyphenols

## Abstract

An anthocyanin-rich diet is considered to protect against chronic inflammatory processes although the bioavailability of anthocyanins is regarded as rather low. Moreover, the immunomodulatory role of anthocyanins is not fully understood yet. In the present study, fractions of blackberry (*Rubus fruticosus*) juice were investigated in plasma-relevant concentrations with respect to their immunomodulatory properties in lipopolysaccharide (LPS)-challenged THP-1-derived macrophages. The complex blackberry extract acted ineffective as well as potential degradation products. Cyanidin-3*O*-glucoside (Cy3glc), the main constituent of blackberry anthocyanins, diminished TNF-α levels at a concentration of 0.02 µg/mL, indicating protective effects as measured with quantitative RT-PCR and multiplex cytokine assays. LPS-boosted activity of transcription factor nuclear factor kappa-light-chain-enhancer of activated B cells (NF-κB) of differentiated THP-1 reporter gene cells was marginally inhibited by Cy3glc. LPS-induced microRNA-155 was further increased, supporting the evidence of protection. Of note, fractions obtained from blackberry juice, in particular cyanidin-3*O*-(6″-dioxalylglucoside), were displaying potential pro-inflammatory properties as these elevated IL-6 and TNF-α levels. In conclusion, highly purified anthocyanin fractions of blackberry juice display both anti- and pro-inflammatory properties at plasma-relevant concentrations depending on their structure and substitution pattern.

## 1. Introduction

Anthocyanins are water-soluble pigments of plants and responsible not only for the red, blue or purple colors of many flowers, but also of a spectrum of fruits and vegetables [1]. An increasing number of studies have indicated that the consumption of anthocyanin-rich fruits and vegetables are inversely associated with inflammatory stress. Thus, a higher intake is considered to result in a lower prevalence of cancer, cardiovascular and Alzheimer’s disease [2,3,4]. In particular, different types of berries such as elder-, blue-, choke- and blackberries represent rich sources of anthocyanins and are well described for their antioxidant properties [5,6].

These studies are mostly limited to the measurement of pro- and anti-inflammatory cytokine gene transcription and secretion [7,8]. However, knowledge is scarce regarding the mechanisms involved in anthocyanin activity, affecting those cytokines. The transcription factor nuclear factor kappa-light-chain-enhancer of activated B cells (NF-κB) represents one of the regulating parameters of cytokine secretion, being indispensable in inflammatory processes and innate immunity. It is a major player in inflammatory signal transduction promoting the expression of more than 100 target genes, most of them involved in host immune response, including pro-inflammatory cytokine interleukin 6 (IL-6), tumor necrosis factor-alpha (TNF-α) or interleukin 8 (IL-8) and the anti-inflammatory interleukin 10 (IL-10) [9,10]. The activation of NF-κB by an inflammatory stimulus is cell-type specific. Selectivity and fine-tuning of the NF-κB-dependent transcriptional response can be achieved by selective activation and binding of different Rel/NF-κB family members to the transcription factor response element. This leads to induction of different target genes [11]. Only a few in vitro studies, addressing the question whether polyphenols affect NF-κB activity, point out the selectivity of the transcription factor. There is evidence that polyphenols, e.g., procyanidin-B_2_ and phloretin may modulate the NF-κB signaling pathway in a way that cytokine gene expression will be decreased, resulting in less cytokine secretion [12,13]. Moreover, an inhibiting effect of cyanidin-3*O*-glucoside (Cy3glc), one of the most studied anthocyanins, on NF-κB activation has been described hinting towards the involvement of the pathway in anthocyanin-mediated immunomodulatory effects [14].

In recent years, there has been growing interest in inflammation-related microRNAs (miRNAs) as regulating factors of cytokine expression. MiRNAs are a class of small noncoding RNAs of 18–24 nucleotides of length known to suppress the expression of protein-coding genes. The impact of miRNAs on inflammatory processes occurs post-transcriptionally and may be mediated by mRNA destabilization and/or translational repression. They are targeting a variety of different mRNAs involved in the regulation of the inflammatory response [15,16]. However, knowledge about their mode of action and targets is still scarce, especially in the context of inflammation. For instance, miR-146 is targeting IL-1R-associated kinase 1 (IRAK-1) and the TNF receptor associated factor 6 (TRAF-6) by decreasing the protein level. Both are important signaling proteins of the canonical NF-κB signaling pathway, which leads into the induction of pro- and anti-inflammatory cytokine gene transcription [17,18]. In turn, other miRNAs like miR-223 and miR-199 are controlling NF-κB activity by targeting NF-κB kinases (IKKs) [19,20]. Additionally, the direct regulation of cytokine mRNA may occur through miRNAs such as miR-125b, which is downregulated during inflammation to guarantee TNF-α production [21]. MiR-125b has been described as an activator of TNF-α cytokine gene transcription during LPS stimulation. It has also been shown that miR-125b might be repressed in order to activate TNF-α gene transcription [21]. In contrast, miR-155 and miR-146a are described as inhibitors of pro-inflammatory cytokine gene transcription, decreasing the mRNA levels of TNF-α, IL-8 and IL-6 [17,18]. MiR-16 has been found to be, either overexpressed or down-regulated in order to decrease or increase TNF-α stability, respectively [22]. Some studies already indicate that different classes of polyphenols out of the class of flavanols, flavonols or stilbenes suppress cytokine gene expression via posttranscriptional regulation [23,24,25]. Quercetin as well as its methoxylated metabolite/analogue isorhamnetin decrease the transcript level of miR-155 significantly at 10 µM after 6 h of incubation in RAW264.7 macrophages. In turn, miR-155 appears to target TNF-α mRNA expression [24]. However, to the best of our knowledge, in the present study the potential impact of blackberry anthocyanins on inflammation-related miRNAs (miR-16, miR-125b, miR-155 and miR-146a) and NF-κB activity in LPS-challenged THP1 macrophages is described for the first time. 

**Figure 1:**
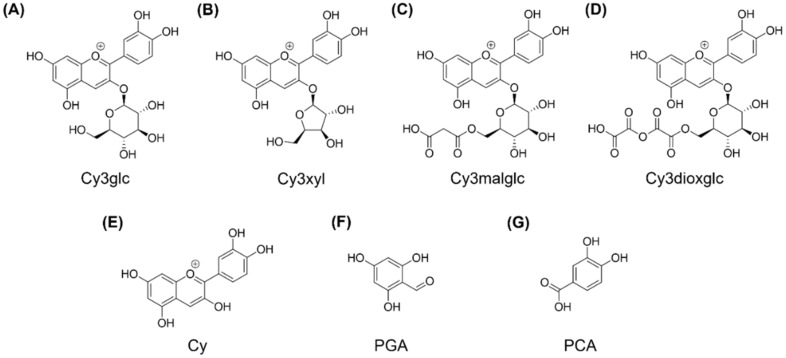
Chemical structures of purified blackberry anthocyanins: (**A**) cyanidin-3*O*-glucoside (*anthocyanin A1*), (**B**) cyanidin-3*O*-xyloside (*anthocyanin A2*), (**C**) Cyanidin-3*O*-(6″-malonylglucoside) (*anthocyanin A3*), (**D**) cyanidin-3*O*-(6″-dioxalylglucoside) (*anthocyanin A4*) and potential degradation products (**E**) cyanidin, (**F**) phloroglucinol aldehyde and (**G**) protocatechuic acid.

Within the scope of the European AnthoPLUS project (www.anthoplus.com; accessed on 6 September 2021), anthocyanins of different complexity in their side chain decoration and sugar moieties were produced and assessed for their quality and properties as potential colorants and food additives. As a novel concept, test kits from different fruit juice sources were developed, so called “AnthoKits”, comprising varying fractions of purified anthocyanins [26]. In the present study, for the first time, an AnthoKit from blackberry (*Rubus fruticosus*) juice was produced and tested. The aim was to investigate the impact of blackberry constituents on LPS-induced pro-inflammatory cytokine transcription and secretion of IL-6, IL-8 and TNF-α in human THP-1 derived macrophages in plasma-relevant concentrations. The AnthoKit comprised an extract of raw blackberry juice (BB juice), four individual purified anthocyanin fractions (A1–A4, Figure 1), a fraction of non-identified polyphenols (n.i. PP) and a mixture of the four fractions A1–A4 based on their natural composition in the blackberry juice. Well-characterized THP-1 monocytes, differentiated by treatment with phorbol-12-myristate-13-actetate (PMA) to adherent macrophages [27], served as an in vitro cell model for intestinal macrophages, the most abundant leukocytes in the intestinal lamina propria, playing a crucial role in the intestinal immune system. One of their main functions seems to be the production of inflammatory mediators [28]. Furthermore, this study intended to elucidate underlying mechanisms leading to inflammatory cytokine production. Hence, the ability of tested anthocyanins and mixtures to modify NF-κB activation as well as the modulation of post-transcriptionally interfering, inflammation-related miRNAs was investigated for the first time to better understand the effect of single anthocyanins in complex mixtures. In vivo, ingested anthocyanins might be already absorbed in the stomach and the small intestine passing rapidly to the blood stream. However, remaining anthocyanins which pass the upper gastrointestinal tract (GIT) represent known substrates for the gut microbiome, giving raise to degradation products such as respective aglycons, phenolic acids and phloroglucinol aldehyde [29]. Systemic bioavailability of anthocyanins is low due to the fact that the glycosides are rapidly hydrolyzed by the gut microbiome [30]. The stability of the liberated aglycons strongly depend on the pH of the environment. Thus, in our study not only the free aglycon cyanidin (Cy) but also the potential degradation products, phloroglucinol aldehyde (PGA) and protocatechuic acid (PCA), were included (Figure 1). 

## 2. Results

### 2.1. Phytochemical Characterization of Blackberry Raw Juice

**Figure 2:**
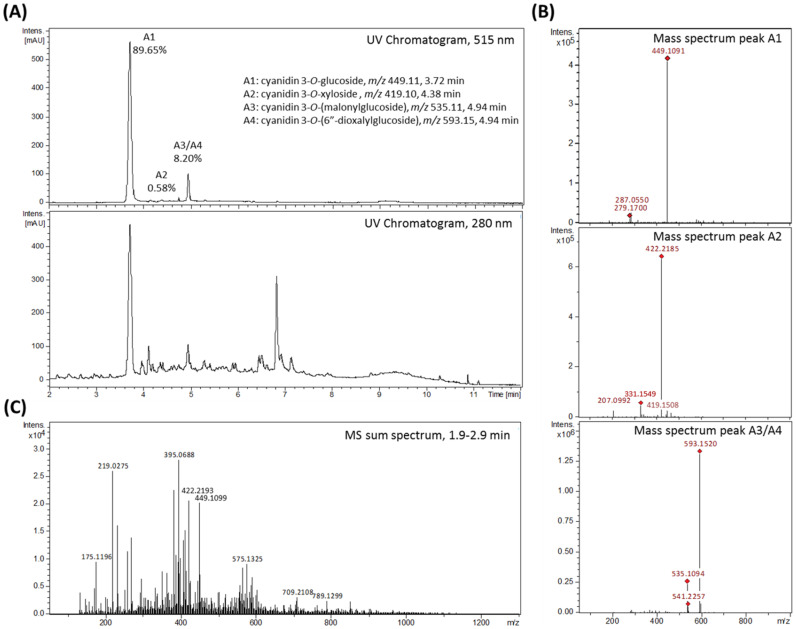
Polyphenolic profile of blackberry raw juice, analyzed by LC–UV/MS. (**A**) UV–chromatograms at 515 nm (top), with percentage of total anthocyanin content calculated from integrated peak areas, and 280 nm (bottom) from 2–12 min. (**B**) Sum of mass spectra from retention time 3.6–3.9 min (anthocyanin A1), 4.35–4.45 min (anthocyanin A2), and 4.9–5.0 min (anthocyanin A3 and anthocyanin A4). (**C**) Sum of mass spectra acquired from MS direct injection, showing all detected masses and their abundances. Tentative annotation based on MS/MS fragmentation pattern (Supplementary Figure S1) and comparison with literature data.

The total anthocyanin content of freshly squeezed blackberry juice has been determined to be 0.25 µg/µL. Based on 400 mL blackberry juice a degreased extract was prepared to reach 100 mg total anthocyanins for subsequent purifications. After lipid removal and filtration, the raw juice sample was analyzed by LC-UV/MS (Figure 2). The UV chromatogram at 280 nm showed a number of chromatographic peaks, likely related to polyphenols naturally present in the blackberry juice (Figure 2A, bottom). In contrast, only two major and six minor chromatographic peaks were observed at 515 nm, the wavelength specific for anthocyanins (Figure 2A, top). The sum of mass spectra across these chromatographic peaks revealed *m/z* 449.11 as the main molecular ion present in peak A1, *m/z* 422.22 in peak A2, and *m/z* 535.11 and *m/z* 593.15 in peak A3/A4 (Figure 2B), the three peaks that are relevant for the finally isolated anthocyanin and polyphenols fractions. Based on MS/MS fragmentation pattern (Supplementary Figure S1) and comparison with literature data the related compounds were tentatively annotated as cyanidin-3*O*-glucoside (*m/z* 449.11, further named anthocyanin A1), cyanidin-3O-(6″-malonylglucoside) (*m/z* 535.11, anthocyanin A3) and cyanidin-3*O*-(6″-dioxalylglucoside) (*m/z* 593.15, anthocyanin A4) (structures Figure 1). The main molecular ion present in peak A2 with *m/z* 422.22 (the major compound of the final polyphenols fraction) could not be annotated so far. However, an additional molecular ion detected in the chromatographic peak A2 was tentatively annotated as cyanidin-3O-xyloside (further named anthocyanin A2) with *m/z* 419.10 (Figure 2A, top, and Supplementary Figure S1B); which could be enriched during anthocyanin isolation procedure. The sum of mass spectra acquired from MS direct injection showed that *m/z* 422.22 and anthocyanin A1 are the fourth and fifth most abundant molecular ions detected in blackberry raw juice, respectively (Figure 2C).

### 2.2. Phytochemical Characterization of Blackberry Anthocyanin Fractions and AnthoKit Mix Preparation

We aim to distinguish bioactive effects of individual anthocyanins from those of the anthocyanin mixture (AnthoKit mix) and other polyphenols as present in blackberry raw juice samples. Thus, fractions of individual anthocyanins have been isolated and a reconstituted AnthoKit mix was prepared thereof as described in materials and methods. The concept is schematically presented in Supplementary Figure S2.

**Figure 3:**
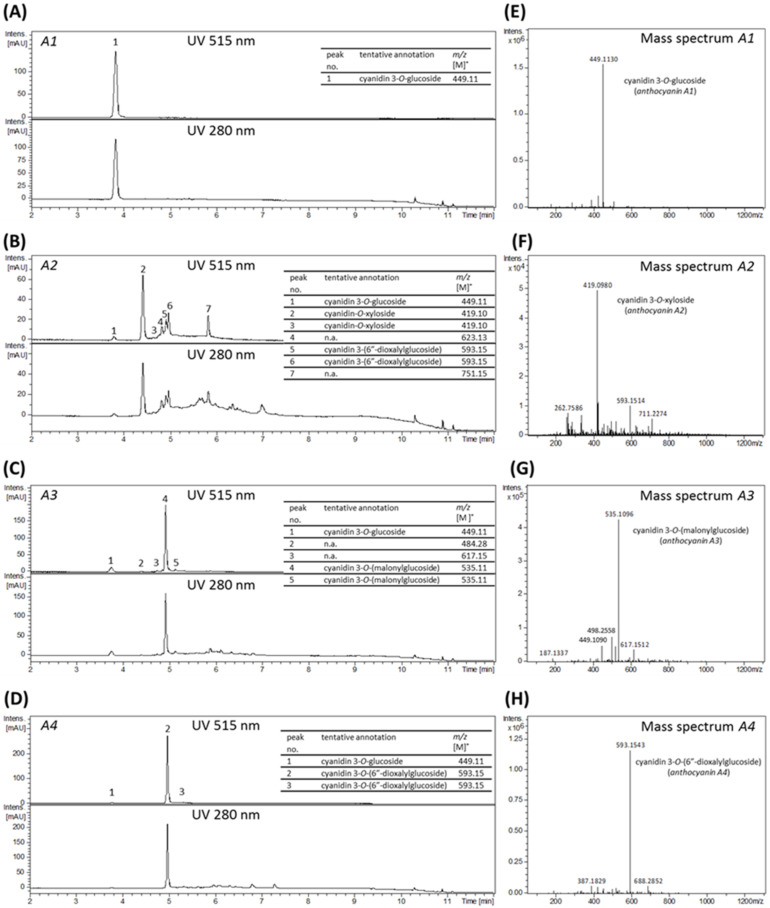
LC-UV/MS analysis of isolated anthocyanin fractions. (**A**–**D**) UV-chromatograms at 515 nm (top) and 280 nm (bottom) from 2–12 min; and (**E**–**H**) sum of mass spectra of fractions anthocyanin A1, anthocyanin A2, anthocyanin A3 and anthocyanin A4, obtained from MS direct injection. Tentative annotation based on MS/MS fragmentation pattern (Supplementary Figure S3) and comparison with literature data; n.a.: not annotated.

Phytochemical compositions of the isolated fractions were analyzed by LC-UV/MS (Figure 3). The fraction anthocyanin A1 showed one prominent chromatographic peak at 515 nm at a retention time of 3.82 min, which has been proven to be enriched for cyanidin-3*O*-glucoside (*m/z* 449.11) as presented in Figure 3A,E. Only this peak at 3.82 min was detected analyzing the data acquired at 280 nm, indicating a high purity of fraction A1 (Figure 3A). For fraction anthocyanin A2 seven anthocyanin peaks were detected, of which peak 2 eluting at 4.41 min contained the main anthocyanin present in this fraction, namely cyanidin-3*O*-xyloside with *m/z* 419.10, while other anthocyanins like cyanidin-3*O*-glucoside and cyanidin-3*O*-(6″-dioxalylglucoside) were only present in minor amounts (Figure 3B,F). Similarly, one major peak was enriched in fraction anthocyanin A3 eluting at a retention time of 4.92 min and corresponding to cyanidin-3*O*-malonylglucoside (*m/z* 535.11), while minor peaks were detected for other anthocyanins, such as cyanidin-3*O*-glucoside (Figure 3C,G). 

The LC-UV chromatogram of fraction anthocyanin A4 revealed one major peak at 515 nm with a retention time of 4.97 min, which is enriched in cyanidin-3*O*-(6″-dioxalylglucoside), as evaluated by MS analysis (Figure 3D,H). Additionally, cyanidin-3*O*-glucoside was detected in this fraction in slight amounts. The tentative annotation of the detected main compounds of each fraction (anthocyanin A1, A2, A3 and A4) was further confirmed based on MS/MS fragmentation pattern (Supplementary Figure S3) and comparison with literature data. Fractions anthocyanin A2, A3, and A4 contained minor remaining amounts of other polyphenols as observed from the UV-chromatograms at 280 nm.

The UV-chromatogram of the polyphenol fraction at 280 nm showed a clear reduction of the peak at a retention time of 3.82 min, which relates to anthocyanin A1 in the profile obtained for the raw juice (Supplementary Figure S4C,D). Other minor differences probably relate to selective isolation and degradation processes during the enrichment procedure. From the sum of mass spectra acquired from MS direct injection we observed one major molecular ion in the polyphenol fraction, being *m/z* 422.22 (Supplementary Figure S4A). MS/MS fragmentation analysis of this molecular ion revealed the release of *m/z* 331.15, a molecular ion mass similar to the one of the anthocyanidin malvidin (Supplementary Figure S4B), but the slight mass difference to the precursor ion does not allow for clear annotation of a malvidin derivative. Furthermore, the molecular ion with *m/z* 422.22 has not been enriched by the anthocyanin specific isolation procedure. The extracted ion chromatograms (EIC) for *m/z* 422.22, revealed two peaks at 4.10 and 4.43 min in the polyphenol fraction as well as in the raw juice fraction (Supplementary Figure S4C,D). This again proves the pre-existence of this compound in the raw juice sample, where it was also detected by means of MS direct injection in major amounts, as the fourth most abundant polyphenolic compound (Supplementary Figure S4E and Figure 2C). Additionally, *m/z* 422.22 could be identified as the main molecular ion in the sum of mass spectra from retention time 4.35–4.45 min, derived from the chromatographic peak A2 at 515 nm (Figure 2B, middle panel). However, the two peaks in the mass chromatograms for *m/z* 422.22 in Supplementary Figure S4C,D are indicative for the presence of two isomeric structures of this compound. Although its UV-vis spectrum is in support with its anthocyanin nature (Supplementary Figure S4B), the mass characteristics of the compound are unusual for an anthocyanin and thus the identity of the main polyphenols component could not be clarified in our study. The above described findings for the molecular ion with *m/z* 422.22 are in agreement with those made for fruits of different strawberry cultivars by da Silva et al. [31].

LC-UV analysis proved the strong enrichment of polyphenols, which is about tenfold, characterized by increased absorbance at 280 nm for the polyphenols fraction, compared to the raw juice one (Supplementary Figure S4C,D). From the UV-chromatogram at 515 nm we concluded minor abundance of anthocyanins (Supplementary Figure S4C, top); mostly cyanidin-3*O*-glucoside at a retention time of 3.82 min. Catechin and epicatechin (*m/z* 291) as well as gallocatechin and epigallocatechin (*m/z* 306) have not been detected in the polyphenol fraction.

By means of plate photometric assays anthocyanin and sugar amounts were determined (Supplementary Table S1). Soluble sugars were nearly completely removed by the chromatographic separations. Levels of glucose, fructose and sucrose were markedly reduced to ≤0.03 mM in all fractions *(anthocyanin A1–A4 and polyphenols). The final fractions (A1, A2, A3, A4 and polyphenols*) contain 49.99 mg, 1.03 mg, 1.34 mg, 3.68 mg and 9.54 mg of anthocyanins, respectively.

To obtain a reconstituted fraction, the AnthoKit mix, we combined the individual purified anthocyanins based on their natural composition in the blackberry juice (Figure 2A,B) and depending on the purity of the final anthocyanin fractions (Figure 3), as described in the materials and methods section. All samples were dried under liquid nitrogen and weighed. We calculated a multiplication factor for the utilization in the biomedical assays according to the anthocyanin concentration estimated by plate photometric assay. All fractions were covered with argon and stored at −20 °C until their use.

In the bioactivity studies abbreviations were chosen based on the characterization of the blackberry fractions as follows: pure blackberry juice extract = BB juice, fraction A1 = Cy3glc, fraction A2 = Cy3xyl, fraction A3 = Cy3malglc, fraction A4 = Cy3dioxglc, mix of fraction A1–A4 = AnthoKit mix; fraction of non-identified polyphenols = n.i. PP.

### 2.3. Impact on Viability of THP-1 Derived Macrophages

To exclude an overlay of immunomodulatory effects with cytotoxicity, potential cytotoxic effects of the blackberry AnthoKit components and respective degradation products (Cy, PCA, PGA) were measured up to a concentration of 5 µg/mL and 10 µM, respectively, by performing the Alamar blue assay [32]. The results indicate no significant toxic effects of all test compounds within the tested concentration range after 20 h of incubation (data not shown). Hence, potential artefacts of the results due to cytotoxicity can be excluded.

### 2.4. Immunomodulatory Effects on LPS-Induced Cytokine Transcript Levels

**Figure 4:**
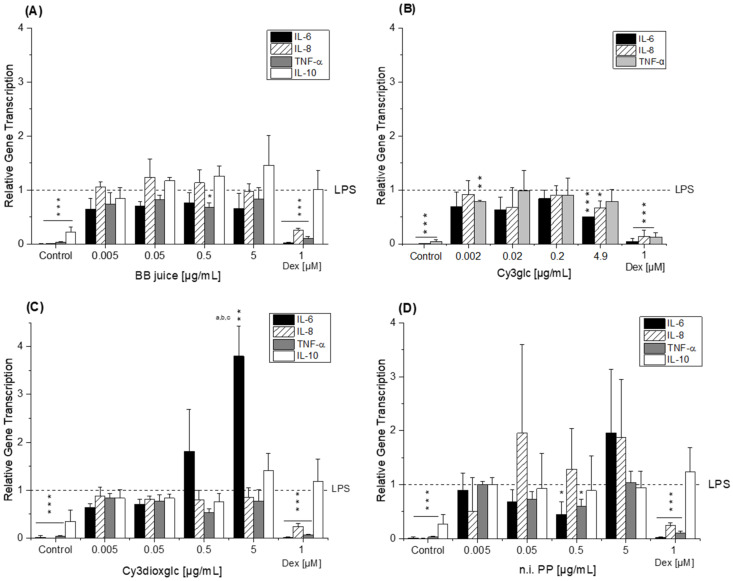
Relative transcript levels of IL-6, IL-8 and TNF-α in THP-1 derived macrophages pre-incubated (2 h) with (**A**) BB juice, (**B**) Cy3glc, (**C**) Cy3dioxglc and (**D**) n.i. PP and subsequently challenged by LPS for another 3 h. Dexamethasone (Dex; 1 µM) served as positive control. BB juice = pure juice extract; Cy3glc = cyanidin-3*O*-glucoside; Cy3dioxglc = cyanidin-3*O*- (6″-dioxalylglucoside); n.i. PP = fraction of non-identified polyphenols. Values shown are the means + SD of at least three independent experiments presented as relative gene transcription, normalized to β-actin and GAPDH and compared to LPS stimulated cells (calibrator, y = 1). Statistical differences were calculated with a one-sample *t*-test (* *p*, ** *p*, *** *p* < 0.05, 0.01, 0.001). Significant differences among the test concentrations were calculated by one-way ANOVA (*p* < 0.05, a–c).

**Figure 5:**
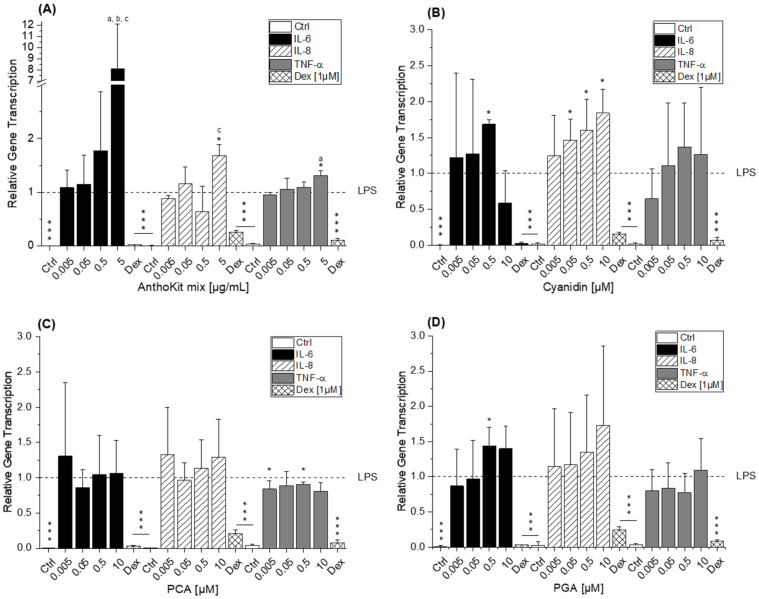
Relative transcript levels of IL-6, IL-8 and TNF-α in THP-1 derived macrophages pre-incubated (2 h) with (**A**) AnthoKit mix, (**B**) cyanidin, (**C**) PCA (protocatechuic acid) and (**D**) PGA (phloroglucinol aldehyde) and subsequently challenged by LPS for another 3 h. Dexamethasone (Dex; 1 µM) served as positive control. AnthoKit mix = mixture comprising all purified anthocyanin fractions based on their natural composition in the blackberry juice. Values shown are the mean + SD of at least three independent experiments presented as relative gene transcription, normalized to β-actin and GAPDH and compared to LPS stimulated cells (calibrator, y = 1). Statistical differences were calculated with a one-sample *t*-test (* *p*, *** *p* < 0.05, 0.001). Significant differences among the test concentrations were calculated by one-way ANOVA (*p* < 0.05, a–c).

THP-1 derived macrophages were pre-incubated (2 h) with blackberry AnthoKit components or respective degradation products and subsequently additionally challenged by LPS for another 3 h to induce transcription of the pro-inflammatory cytokines TNF-α, IL-6, and IL-8 as well as of the anti-inflammatory IL-10 cytokine. LPS potently induced cytokine transcription in THP-1 derived macrophages compared to control cells (Figure 4 and Figure 5) as transcript levels in non-stimulated macrophages were significantly lower compared to levels in LPS-stimulated cells (*p* ≤ 0.001), which were used as calibrator and thus set to 1. Dexamethasone (Dex, 1 µM) served as positive control due to its potent anti-inflammatory properties. LPS-induced IL-6, IL-8 and TNF-α transcript levels were potently reduced by dexamethasone by 75–95%, (*** *p* ≤ 0.001), whereas IL-10 transcript levels were not affected (Figure 4 and Figure 5).

The BB juice did not affect the transcript levels of IL-6, IL-8 and IL-10 up to 5 µg/mL. Only the relative quantity of TNF-α mRNA was reduced to 0.7 ± 0.1-fold by 0.5 µg/mL BB juice with statistical significance (Figure 4A). Cy3glc significantly decreased IL-6 and IL-8 transcript levels at 4.9 µg/mL (equals 10 µM) compared to non-treated LPS-stimulated cells (Figure 4B). TNF-α cytokine mRNA was reduced by Cy3glc already at a concentration of 0.002 µg/mL (equals 0.005 µM; *p* ≤ 0.01). Cy3xyl and Cy3malglc were not effective in reducing cytokine mRNA levels, hence possessing no modulatory activity against the LPS-induced inflammatory response of THP-1 derived macrophages (Supplementary Figure S5). In contrast, Cy3dioxglc further increased IL-6 cytokine mRNA at 0.5 and 5 µg/mL with statistical significance up to 3.8 ± 0.6-fold (Figure 4C), suggesting rather pro-inflammatory properties of this blackberry anthocyanin. However, a tendency towards reduction of TNF-α transcript levels to 0.5 ± 0.1-fold at a concentration of 0.5 µg/mL was also evident. Likewise, the n.i. PP fraction significantly decreased the amount of TNF-α and IL-6 mRNA at a concentration of 0.5 µg/mL but caused rather additional increase in IL-6 and IL-8 transcription at the highest concentration tested (Figure 4D).

Applying the AnthoKit mix, comprising all purified anthocyanin fractions combined based on their natural composition in the BB juice, the cytokine transcript levels of IL-6, IL-8 and TNF-α were additionally increased in LPS-challenged THP1-derived macrophages at the highest test concentration (Figure 5A). IL-6 mRNA was 8.1 ± 4.0-fold higher in macrophages incubated with 5 µg/mL AnthoKit mix than in LPS-stimulated cells, IL-8 mRNA 1.7 ± 0.2-fold and TNF-α 1.3 ± 0.1-fold. 

The suppression of IL-6 levels by the main anthocyanin Cy3glc was lost, an unexpected result, considering the 90% portion of Cy3glc in the AnthoKit mix. However, it nicely reflects potential interactions in mixtures, leading to a non-predictable outcome. Another important aspect in this context could be the production of bioactive degradation products exhibiting diverse activities compared to the parent compound. Therefore, we included Cy, PCA and PGA in our testing.

Of note is the rather pro-inflammatory potential of Cy and PGA as incubation lead to further significant increase of LPS-induced IL-6 and, in particular, IL-8 mRNA levels at 0.5 µM (Figure 5B,D). Cy and PGA had no impact on TNF-α cytokine gene transcription, only PCA marginally yet significantly reduced TNF-α transcript levels (Figure 5B–D) leading to the conclusion that the degradation products only marginally contribute to anti-inflammatory effects in challenged THP-1 derived macrophages. However, this might promote the hypothesis that degradation products contribute to the elevated levels of pro-inflammatory cytokine mRNA provoked by the blackberry AnthoKit mix. Furthermore, potential interactions between anthocyanins of the mixture might affect their stability differently and thus, their bioactivity. 

### 2.5. Immunomodulatory Effects on LPS-Induced Cytokine Secretion

**Figure 6:**
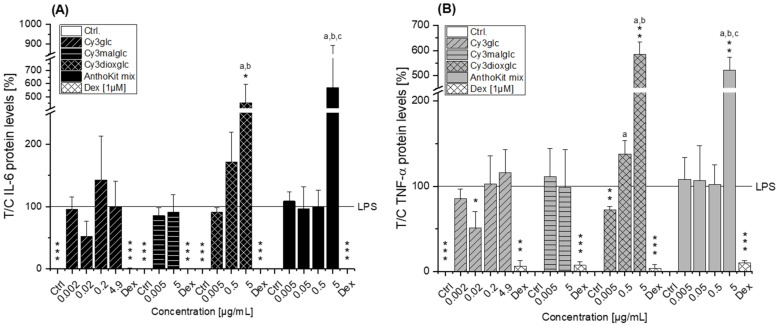
Release of (**A**) IL-6 and (**B**) TNF-α from THP-1 derived macrophages pre-incubated (2 h) with Cy3glc, Cy3malglc, Cy3dioxglc and AnthoKit mix and subsequently challenged by LPS for another 18 h. Dexamethasone (Dex; 1 µM) served as positive control. AnthoKit mix = mixture comprising all purified anthocyanin fractions based on their natural composition in the blackberry juice. Values plotted are the mean + SD of three independent experiments and presented as test over control compared to LPS stimulated cells. Significant differences among the test concentrations were calculated with one-way ANOVA (*p* < 0.05, a–c). Statistical differences compared to the LPS stimulus were calculated with a one-sample *t*-test (* *p*, ** *p*, *** *p* < 0.05, 0.01, 0.001). No statistical evaluation could be performed for the solvent controls in 5B since measured TNF-α levels equaled 0 ng/µL.

Whether the modulatory effect on inflammatory cytokine transcript levels is mirrored at the secretion level was investigated by applying a magnetic bead-based multiplex immunoassay and representative anthocyanins and degradation products. Cy3glc, appearing to have potential anti-inflammatory properties, Cy3dioxglc, AnthoKit mix and PGA, showing rather inducing-effects with respect to cytokine transcription and Cy3malglc showing no effects, were tested. IL-6 and TNF-α cytokine release was measured in THP-1 derived macrophages after 2 h of pre-incubation followed by 18 h of additional LPS stimulation to allow cytokine secretion. IL-6 and TNF-α cytokine release from non-stimulated cells was significantly different compared to LPS stimulated cells (*** *p* ≤ 0.001, Figure 6). The positive control Dex potently suppressed IL-6 and TNF-α release by 93.6–99.9% (** *p*,*** *p* ≤ 0.01, 0.001). As shown in Figure 6A, Cy3glc had no impact on LPS-induced IL-6 cytokine release. At the concentration of 0.02 µg/mL (0.05 µM) Cy3glc a reduction of IL-6 is indicated, yet not reaching statistical significance.

In contrast, Cy3glc significantly decreased the secretion of TNF-α cytokine at 0.02 µg/mL compared to LPS stimulation (* *p* ≤ 0.05), suggesting an anti-inflammatory effect (Figure 6B). As expected, the non-effective Cy3malglc had no impact on either IL-6, nor on TNF-α release at 0.005 and 5 µg/mL (Figure 6A,B). Intermediate concentrations of Cy3malglc did also not show any effect but could not be evaluated statistically as compound limitations did not allow sufficient replicate measurement. Moreover, the rather increasing effect of PGA on IL-6 compared to the LPS effect observed at the RNA level was not reflected at the cytokine secretion level. PGA rather seemed to decrease the LPS-induced secretion of IL-6 at all concentrations tested, yet without statistical significance (Supplementary Figure S6). The in first instance pro-inflammatory Cy3dioxglc and AnthoKit mix also potently increased the release of IL-6 4.6 ± 1.4 and 5.7 ± 3.2-fold and of TNF-α 5.8 ± 0.5 and 5.2 ± 0.5-fold, respectively, in LPS-stimulated THP-1-derived macrophages at the highest concentration applied, thereby strengthening the assessment to act pro-inflammatory. However, at the lowest concentration of 0.005 µg/mL Cy3dioxglc significantly decreased LPS-induced TNF-α secretion (Figure 6B).

### 2.6. Modulatory Effects on NF-κB Activity

**Figure 7:**
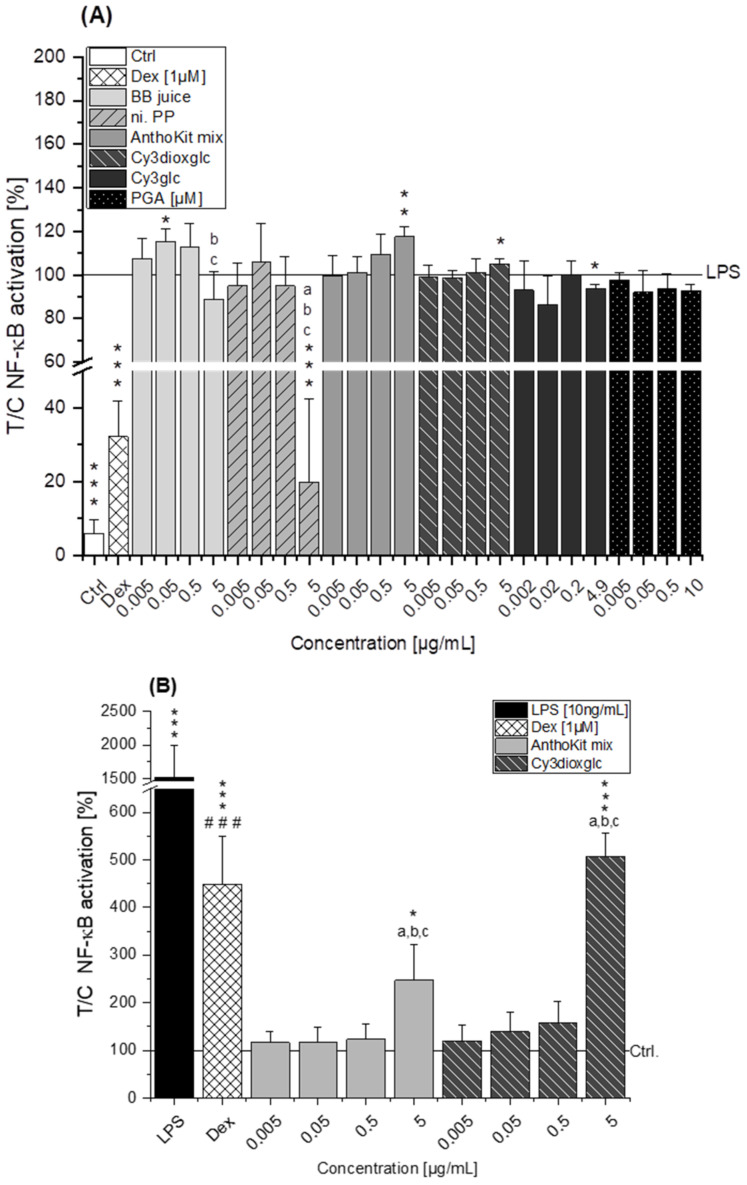
Modulation of NF-κB activity by (**A**) BB juice, n.i. PP, AnthoKit mix, Cy3dioxglc, Cy3glc and PGA pre-incubated for 2 h and subsequently additionally stimulated with LPS for 18 h and (**B**) AnthoKit mix and Cy3dioxglc incubated for 20 h in differentiated THP1-Lucia™ NF-κB cells expressing the NF-κB-regulated reporter gene luciferase. Dexamethasone (Dex; 1 µM) served as positive control. BB juice = pure blackberry extract; n.i. PP = fraction of non-identified polyphenols; AnthoKit mix = mixture comprising all purified anthocyanin fractions based on their natural composition in the blackberry juice; Cy3dioxglc = cyanidin-3*O*-(6″-dioxalylglucoside); Cy3glc = cyanidin-3*O*-glucoside; PGA = phloroglucinol aldehyde. Values shown are the mean + SD of at least three independent experiments and presented relative to the LPS stimulus or the solvent control (=100%). Significant differences among the test concentrations were calculated with one-way ANOVA (*p* < 0.05, a–c). Statistical differences compared to the respective control (100%) were calculated with a one-sample *t*-test (* *p*, ** *p*, *** *p* < 0.05, 0.01, 0.001) or with a two-sample *t*-test between LPS and Dex (### *p* < 0.001).

As NF-κB is a key transcription factor of M1 macrophages, it is required for the regulation of pro-inflammatory cytokines such as IL-6 and TNF-α. To address the question whether it is a potential target for blackberry anthocyanins, the impact of the BB juice, n.i. PP, AnthoKit mix, Cy3dioxglc, Cy3glc and PGA on the activity of NF-κB was investigated by treating stable transfected THP-1 cells with the NF-κB-regulated reporter gene luciferase. Cells were differentiated to macrophages, pre-incubated for 2 h with the test substances and additionally stimulated with LPS for 18 h, resulting in a potent increase of luciferase activity indicative for NF-κB activation. Non-stimulated cells and Dex-treated cells (1 µM, positive control) exhibited significant lower NF-κB activity in comparison to LPS stimulated cells (NF-κB activity reduction: 94% and 68%, respectively; *** *p* ≤ 0.001, Figure 7A). 

Pre-incubation with the degradation product PGA (up to 10 µM) did not affect LPS-induced NF-κB activity (Figure 7A). Cy3glc instead (4.9 µg/mL) marginally decreased luciferase expression in comparison to LPS with statistical significance (* *p* ≤ 0.05), indicating suppression of the activated signaling pathway (Figure 7A). In contrast, Cy3dioxglc as well as the AnthoKit mix significantly increased luciferase expression in LPS-challenged macrophages of stable transfected THP-1 cells at the highest concentration tested, suggesting further NF-κB activation. This added activity potentially explains the increase of IL-6 and TNF-α cytokines observed after incubation with both fractions. The fraction of non-identified polyphenols significantly suppressed luciferase expression at 5 µg/mL down to 20 ± 22%. An unexpected result, as n.i. PP showed only marginal effects on cytokine transcription. Furthermore, a significant increase of NF-κB activation could be observed with 0.05 µg/mL BB juice. However, at 5 µg/mL BB juice led to a non-significant reduction of NF-κB activity.

To further investigate the rather pro-inflammatory effects observed by incubation with the AnthoKit mix and Cy3dioxglc, these substances were incubated for 20 h without LPS stimulation. Cells incubated with LPS (18 h, 10 ng/mL) or pre-incubated with Dex for 2 h and subsequently additionally challenged with LPS for 18 h served as controls. LPS significantly induced NF-κB activity in Lucia™ NF-κB macrophages when compared to the solvent control (*** *p* < 0.001, Figure 7B). Additionally, Dex significantly reduced the LPS-induced NF-κB activation (### *p* < 0.01). In cells not challenged with LPS, the AnthoKit mix as well as Cy3dioxglc provoked a significant NF-κB activation up to 2.5 ± 0.8 and 5.1 ± 0.5-fold, respectively, at the highest applied test concentration of 5 µg/mL (Figure 7B). This further points towards a pro-inflammatory mode of action of Cy3dioxglc and the AnthoKit mix.

### 2.7. Impact on Inflammation-Related miRNA Levels

In order to determine whether cellular levels of selected miRNAs involved in LPS-induced inflammatory response are modulated by blackberry anthocyanins or the degradation product PGA, the miRNA transcript levels of miR-16, miR-125b, miR-155 and miR-146a were determined by qRT-PCR. THP-1 derived macrophages were treated randomly for short term (2 h pre-incubation and 3 h LPS) and for long term (2 h pre-incubation and 18 h LPS) to identify the proper timeframe for detection of miRNA-modulation. Short term incubation turned out to be not sufficient (data not shown) as has been also described by Chanput et al. [33]. 

**Figure 8:**
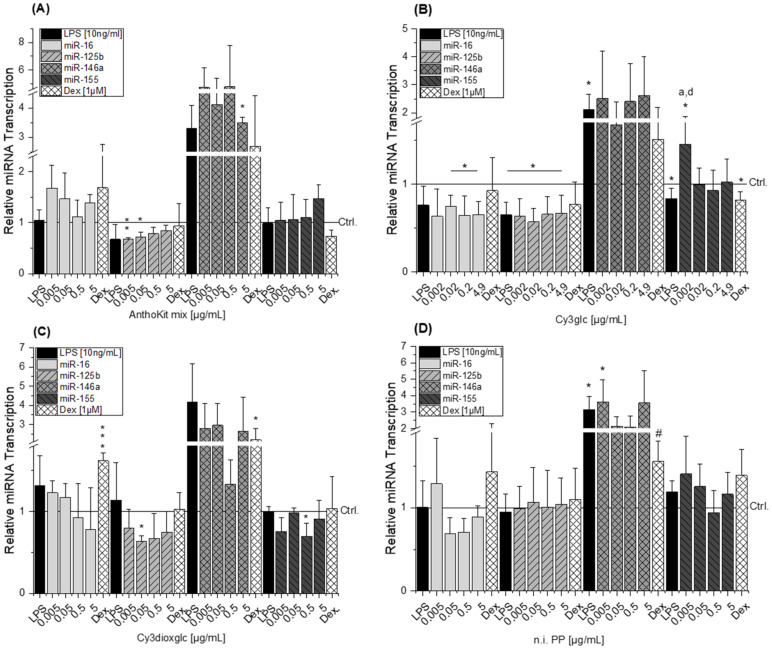
miRNA levels of miR-16, miR-125b, miR-146a and miR-155 in THP-1 derived macrophages pre-incubated with (**A**) AnthoKit mix; (**B**) Cy3glc; (**C**) Cy3dioxglc and (**D**) n.i. PP for 2 h and subsequently additionally challenged with LPS (10 ng/mL). Dexamethasone (Dex, 1 µM) served as a positive control. Cy3glc = cyanidin-3*O*-glucoside; Cy3dioxglc = cyanidin-3*O*-(6″-dioxalylglucoside); AnthoKit mix = mixture comprising all purified anthocyanin fractions based on their natural composition in the blackberry juice; n.i. PP = fraction of non-identified polyphenols. Values are the mean + SD of at least 3 independent experiments presented as relative gene transcription normalized to RNU6 and SNORD68 and compared to the solvent control (Ctrl., calibrator, y = 1). Significant differences among the test concentrations were calculated by one-way ANOVA (*p* < 0.05, a–e). Statistical differences compared to the solvent control or the LPS stimulus were calculated with a one-sample *t*-test (* *p*, ** *p*, *** *p* < 0.05, 0.01, 0.001) or a two-sample *t*-test (# *p* < 0.05), respectively.

LPS-stimulation of THP-1 derived macrophages did not significantly affect miR-16 and miR-155 transcription. Only a minor, yet significant, decline of miR-155 was detected in the data set of Cy3glc. LPS slightly reduced miR-125b levels, however without statistical significance (Figure 8). In contrast, LPS-stimulation led to an increase of miR-146a by more than 2-fold, with statistical significances observed in the data set of Cy3glc and n.i. PP (* *p* < 0.05; Figure 8B,D). miR-16 levels were not significantly affected by the tested compounds. Results regarding the effect of Cy3glc potentially indicate a reducing effect on miR-16 levels as statistics indicate a significant difference between 0.02 and 4.9 µg/mL (Figure 8B). 

A marginal rather reducing tendency in miR-16 levels could be observed with increasing concentrations of Cy3dioxglc (Figure 8C). Increasing concentrations of Cy3glc and n.i. PP did not significantly affect LPS-induced miR-146a levels (Figure 8B,D). Similar results were observed with the data set of PGA (Supplementary Figure S7). At the lowest concentration, the AnthoKit mix and the n.i. PP showed tendencies of further miR-146a induction compared to the LPS stimulus, whereas Cy3dioxglc showed a reducing tendency at 0.5 µg/mL (Figure 8A,C,D). Pre-incubation of THP-1 derived macrophages with the AnthoKit mix, Cy3dioxglc, n.i. PP (Figure 8) and PGA (Supplementary Figure S7) had no significant impact on miR-155 transcription as the miRNA-levels remained unchanged compared to levels of the solvent control. However, Cy3glc significantly induced miR-155 at a concentration of 0.002 µg/mL compared to the LPS stimulus, whereas Cy3dioxglc showed a reducing tendency at 0.5 µg/mL.

## 3. Discussion

In the present study the modulatory effect of blackberry anthocyanins and respective degradation products on LPS-induced pro-inflammatory cytokines IL-6, TNF-α and IL-8 was investigated, to our knowledge, for the first time at physiologically relevant concentrations (0.002–5 µg/mL) in THP-1 derived macrophages. Furthermore, the impact on NF-κB activation and miRNA modulation of the single substances and complex mixtures were investigated.

Corroborated by existing literature [34,35], detected and purified anthocyanins in the blackberry extract were all cyanidin-based. None of the test compounds showed cytotoxic effects after 20 h of incubation in THP-1 derived macrophages (data not shown). This confirms previous findings in literature, where anthocyanins and their degradation products did not show any cytotoxic effects up to 50 µM in THP-1 cells [8].

The blackberry raw juice extract, BB juice, decreased LPS-induced TNF-α transcript levels at 0.5 µg/mL and showed reducing tendencies on IL-6 transcripts (Figure 4A). NF-κB activity was marginally affected by BB juice as it was significantly increased at 0.05 µg/mL but showed a decreasing tendency at 5 µg/mL. This reducing trend can potentially be attributed to the polyphenols present in the extract since the fraction of n.i. PP could significantly downregulate LPS-induced IL-6 and TNF-α transcripts at 0.5 µg/mL and showed a potent inhibition of LPS-induced NF-κB activity at 5 µg/mL. At this point, however, cytotoxic effects on THP1-Lucia™ NF-κB cells cannot be excluded completely as cytotoxicity was only ruled out in THP-1 derived macrophages. Paur et al. reported inhibition of LPS-induced NF-κB activity by blackberry at 100 mg/mL in U937–3κκB-LUC cells after 30 min pre-incubation and additional challenge for 6 h [36]. Albeit the conditions and experimental set-up are different to our experiments the data support our findings, which showed moderate tendencies towards NF-κB inhibition due to the much lower concentrations applied. 

Compared to the non-effective blackberry anthocyanins, Cy3xyl and C3malglc, the main anthocyanin Cy3glc significantly decreased IL-6 and IL-8 mRNA levels (Figure 3B) accompanied with lower IL-6 protein levels (Figure 6). Remarkably, the TNF-α transcript level was already significantly reduced at 0.002 µg/mL Cy3glc, TNF-α protein was diminished at 0.02 µg/mL. This concurs well with previous findings of Triebel et al. [7], showing that Cy3glc has a similar pattern with respect to the effect on pro-inflammatory mediators in human colon T84 cells. However, the cells of this in vitro study were stimulated with a mixture of different cytokines instead of LPS and the applied test-concentrations of Cy3glc were obviously higher for incubation of the epithelial cell line (25–100 µM) than for the stimulated macrophages. Moreover, Cy3glc marginally yet significantly decreased LPS-induced NF-κB activation at 4.9 µg/mL (equals 10 µM), corresponding well with previous literature. Ferrari et al. showed that pre-treatment of Caco-2 cells with 20 and 40 µM Cy3glc could keep TNF-α induced NF-κB and IL-6 induction at solvent control levels [14]. Furthermore, it could be demonstrated that Cy3glc is decreasing IκBα phosphorylation in THP-1 macrophages [37]. Thus, Cy3glc is able to protect cells from inflammatory cytokine release. In fact, contrary to these results di Gesso et al. reported no impact of Cy3glc on NF-κB activity in THP-1 monocytes [8]. This difference potentially reflects the discrepancy between monocytes and macrophages or is based on the shorter incubation time used. Considering that 90% of the anthocyanin fraction comprised Cy3glc, the non-protective effect of the AnthoKit mix is surprising, yet reflects potential interactions of single constituents as has been observed for the BB juice.

Of note, the most effective blackberry anthocyanin Cy3dioxglc additionally increased the level of IL-6 and TNF-α cytokines, suggesting pro-inflammatory properties which are supported by the minor yet significant increase of LPS-induced NF-κB activity with the highest test concentration applied. Furthermore, Cy3dioxglc alone was found to potently activate NF-κB at 5 µg/mL (Figure 7), underpinning pro-inflammatory properties. To the best of our knowledge, the implication of Cy3dioxglc in inflammatory processes has not been described before. The AnthoKit mix, a mixture of the tested anthocyanins comprising 90% of Cy3glc and only 7% of Cy3dioxglc, still additionally elevated IL-6 and TNF-α secretion (Figure 6), potentially intensifying the pro-inflammatory response to LPS. A similar picture was observed with NF-κB activation as 5 µg/mL AnthoKit mix could significantly increase it. This hints towards Cy3dioxglc being the potential key driver of the rather pro-inflammatory mode of action of the AnthoKit mix although only 7% of Cy3dioxglc are contained. Aboonabi et al. could show that 50 µg/mL purified anthocyanins from black currant and bilberry could reduce LPS-induced IL-6 production in primary human diabetic aortic endothelial cells [38]. However, no Cy3dioxglc is to be expected in these extracts since black currant mainly contains anthocyanin-rutinosides and bilberry beholds a complex mixture of anthocyanins comprising predominantly delphinidin-3*O*-glucoside and Cy3glc [39,40]. Van de Velde et al. reported significantly lower IL-6 transcript levels when incubating LPS-challenged RAW 264.7 macrophages with a crude blackberry extract and an anthocyanin- or polyphenol-enriched fraction thereof. However, the transcription of the inflammatory cytokine IL-1β could only be reduced by both of the enriched fractions whereas the crude extract showed significant induction [41]. 

The aglycon Cy showed a significant pro-inflammatory tendency as well, considering the elevated IL-6 and IL-8 transcript levels after 5 h of incubation (Figure 5B). In contrast, PCA, a potential degradation product, yielded a similar decreasing effect on TNF-α transcript levels as observed for Cy3glc. These findings differ from previous results of di Gesso et al. [8], reporting that TNF-α mRNA was slightly increased by PCA treatment at 1 µM after 2.5 h of incubation in THP-1 monocytes. Interestingly, di Gesso and colleagues noted already after 3.5 h of incubation a slight decrease of TNF-α cytokine release with the test substance. Thus, it is quite conceivable that after 5 h of incubation the transcript level of TNF-α might have dropped even more. PGA, typically found in the colon, did not affect the inflammatory response as much as initially expected based on the significant increase of IL-6 mRNA in LPS-induced THP-1 derived macrophages (Figure 5D). However, no impact on either cytokine secretion or NFκB activity was evident. Earlier findings by Kropat et al. have shown, that PGA activated the Nrf2 transcription factor in a Chinese hamster ovary (CHO) cell-based reporter gene assay. It also induced the transcription of anti-oxidative Nrf2 dependent gene haeme oxygenase 1 (HO-1) in HT29 colon carcinoma cells [42]. As a logical consequence, pro-oxidative properties, which are often linked to inflammatory properties, were attributed to the final degradation product PGA. However, the apparent lack of consistency in the case of PGA may be the reflection of an overlay with mechanisms other than NF-κB-mediated fine tuning. For instance, inflammation-related miRNAs affecting cytokine secretion levels post-transcriptionally. 

MicroRNAs have been reported as molecular targets of polyphenols [43,44,45], underlining their capacity to interfere in biological processes. All miRNAs tested are targeting IL-6, IL-8 and TNF-α mRNA. Either by direct mRNA-binding in presence of RNA-binding proteins or by targeting other signaling molecules involved in signal transduction such as myeloid differentiation primary response 88 (MyD88), inhibitor of nuclear factor kappa B kinase subunit epsilon (IKKε) or TRAF-6 [15,46,47,48]. With respect to the response of THP-1 derived macrophages to LPS, the results on miRNAs predominantly correspond to the literature. Transcript levels of miR-146a in non-stimulated THP-1 cells were significantly lower than in LPS-stimulated cells, indicating an up-regulation of miR-146a levels in response to LPS. These findings are in line with previously reported results by Taganov et al. who identified miR-146a as one of several endotoxin-responsive genes in THP-1 cells [18]. However, Dex was not able to convincingly suppress miR-146a with statistical significance in all experimental setups as limited amounts of test compounds did not allow sufficient biological replicates. Furthermore, miR-146a has previously been reported to negatively regulate the translation of IL-6 [49]. This corresponds well with the observed reducing tendency of 0.5 µg/mL Cy3dioxglc which occurs concomitantly with the induction of IL-6 protein levels. However, for the highest concentration of Cy3dioxglc and the AnthoKit mix the effect on miR-146a could not be observed despite the potent induction of IL-6 suggesting that other posttranscriptional regulatory mechanisms are involved in stabilizing IL-6 translation. Tili et al. reported decreased levels of miR-125b in LPS-stimulated murine RAW 264.7 macrophages in comparison to non-stimulated cells. Contrary to that,, they observed an up-regulation of miR-155 [21], which was not the case for the THP-1 derived macrophages in the present study (Figure 7). However, there is still considerable ambiguity with regard to the role of miR-155. A number of studies have demonstrated that both inhibition and overexpression of miR-155 is negatively regulating the expression of cytokines, e.g., IL-1β or IL-8 [50,51]. On the other hand, miR-155 seems to be essential for the expression of TNF-α and IL-6. Indeed, some scientific reports have demonstrated that miR-155 is increased in response to inflammatory processes at an early stage in order to enhance TNF-α production [25,52]. Nevertheless, Nahid et al. found that miR-155 transcript levels are rarely affected during LPS stimulation in THP-1 derived macrophages, whereas TNF-α transcript levels and cytokine release were potently induced [17]. Taken together, the findings by former investigations are contradictory.

Boesch-Saadatmandi et al. investigated the impact of the flavonols quercetin and isorhamnetin on LPS-induced TNF-α transcription as well as cytokine release in RAW 264.7 macrophages after 2 and 24 h of incubation, respectively [24]. The results show that quercetin and isorhamnetin decrease TNF-α mRNA and cytokine level, respectively. Additionally, they studied whether miR-155 transcript levels are altered after 6 h of incubation considering the potential implication of the miRNA in regulating TNF-α expression. The obtained data indicate that both flavonols antagonize LPS-induced miR-155 transcript levels. The authors suggest that the decrease of miRNA transcript level may relate to the inhibition of NF-κB activation and thus, result in the reduction of pro-inflammatory TNF-α. However, our results do not support the observations regarding the impact of bioactive food constituents on miR-155 transcription, although, Cy3glc reduces pro-inflammatory cytokine expression (Figure 3B and Figure 5) and NF-κB activation (Figure 6A). This is not exceptional considering incubation time and concentration of an inflammatory stimulus as crucial factors in influencing the expected response. On the one hand, the observed increase of miR-155 after 20 h of incubation with Cy3glc might thus be considered as a response to the continuous repression of pro-inflammatory cytokine mRNA. On the other hand, other targets of miR-155 include several upstream signaling molecules of the NF-κB pathway, such as MyD88, IKKε or Tak1-binding protein 2 (TAB2) [15,21]. As a result, the observed induction of miR-155 after Cy3glc incubation potentially blocks NF-κB pathway activation leading to suppressed cytokine expression. The results demand further evaluation for fully understanding how the anthocyanin Cy3glc affects miRNA levels. The impact of Cy3dioxglc is even more complex and puzzling as it reduces miR-125b and miR-155 at a concentration of 0.05 or 0.5 µg/mL, respectively, and hence, is signaling protective effects against LPS. However, this contradicts the further activation of LPS-induced NF-κB activation and IL-6 transcription and secretion observed with Cy3dioxglc. 

Taken together, plasma-relevant concentrations of blackberry anthocyanins display different and partly controversial modulatory effects regarding the inflammatory response of THP-1 derived macrophages to LPS. As the blackberry extract, comprising all tested anthocyanins, had no major impact on the cellular stress response, it nicely shows that these effects are well balanced in a mixture. The study also demonstrates that the bioactivity of anthocyanins is not always associated with anti-inflammatory properties. Depending on the composition and the applied concentration also pro-inflammatory effects might occur. It might further be speculated whether these, at least in the present study, limited pro-inflammatory effects are contributing to some hormesis-like effects. However if so, the pro-inflammatory nature of respective bioactive constituents should be taken into account when fortified preparations such as food supplements for regular human consumption are provided.

## 4. Materials and Methods

### 4.1. Chemicals and Plant Material

Cyanidin-3*O*-glucoside chloride (Cy3glc) and cyanidin chloride (Cy) were purchased from Extrasynthése (Lyon, France). Protocatechuic acid (PCA) and phloroglucinol aldehyde (PGA) were obtained from Carl Roth (Karlsruhe, Germany) and Sigma Aldrich (Munich, Germany), respectively. All used compounds had a purity ≥ 95% (HPLC). Stock solutions of the test compounds were prepared at concentrations of 0.005, 0.05, 0.5 and 10 mM in ethanol (acidified, 80% EtOH). Phorbol 12-myristate 13-actetate (PMA; TLC purity ≥ 99%, PMA), lipopolysaccharides from *Escherichia coli* (LPS) and dexamethasone (Dex) were purchased from Sigma Aldrich and were dissolved in DMSO, cell culture medium and EtOH (80%, *v*/*v*), respectively. Freshly squeezed blackberry juice without any further processing was obtained from Bayernwald Früchteverwertung KG (Hengersberg, Germany). The juice was frozen and stored at −80 °C until further use.

### 4.2. Anthocyanin Quantification by UV/Vis Spectral Photometric Detection

Total anthocyanin content was quantified by means of the pH differential method described by Giusti and Wrolstad [53]. In brief, the absorbance of pH = 1.0 (25 mM KCl) and pH = 4.5 (0.4 M Na-acetate) buffer-diluted samples was measured at 515 nm and 700 nm with a SPECTRAMAX^TM^ Pro UV-visible spectrophotometer (Molecular Devices, Sunnyvale, CA, USA). Measurements were done in duplicates. The differential absorbance was calculated using the following equation: A = (A515–A700) pH 1.0—(A515–A700) pH 4.5. The total anthocyanin content was then calculated based on reference curves for cyanidin-3*O*-glucoside in the range from 0.001 mg/mL to 0.05 mg/mL. 

### 4.3. LC-MS Analysis

Prior to injection samples were diluted with solvent A (water + 0.5% formic acid, FA) in a ratio of 1:5 and incubated one hour at 4 °C to allow formation of potential precipitates. After centrifugation the clear supernatant was transferred to a sample vial and 3 µL of this mixture were injected per sample. 

LC-UV/MS profiling was carried out using a combination of an ultra-performance liquid chromatography (UPLC) instrument (H-class, Waters, Milford, MA, USA) with photodiode array (PDA) detection coupled to an ultra-high-resolution time of flight mass spectrometer (UHR-TOF–MS, maXis Impact, Bruker Daltonics, Bremen, Germany) for MS detection. LC separation was based on a method described previously [54]. For MS detection, the outlet of the PDA detector was coupled to the electrospray ionization (ESI) source (positive ion mode at 260 °C dry temperature, 3 bar nebulizer gas pressure, 4000 V capillary voltage and a dry gas flow of 11 L/min) of the UHR-TOF-MS instrument using a splitter directing 83% of the eluate into the source. The instrument was operated in MS full scan mode at 3 Hz acquisition speed using the following settings: mass range *m/z* 50–1300, hexapole radio frequency (RF) voltage 800 V peak-to-peak (Vpp), collision energy 8 eV, funnel 1 RF of 300 Vpp, a funnel 2 RF of 600 Vpp, prepulse storage time 5 µs, transfer time 50 µs, collision cell RF of 500 Vpp. Instrument calibration was done with 10 mM sodium formate solution (12.5 mL H_2_O, 12.5 mL isopropanol, 50 µL concentrated FA, and 250 µL 1M NaOH). 

For LC-MS/MS fragmentation analyses of the blackberry raw juice the instrument was operated in MS full scan mode as indicated above and with auto-MS/MS scan mode activated. MS fragmentation was realized by collision-induced dissociation (CID) with argon at 1.5 mTorr in the quadrupole collision cell of the instrument using the following settings: molecular mass 300 Da (width 5 Da) with 27, 22, and 17 eV for charge state 1, 2, and 3 respectively; molecular mass 500 Da (width 6 Da) with 32, 27, and 22 eV for charge state 1, 2, and 3 respectively; molecular mass 1000 Da (width 8 Da) with 45, 40, and 35 eV for charge state 1, 2, and 3 respectively; molecular mass 1500 Da (width 10 Da) with 45, 40, and 35 eV for charge state 1, 2, and 3 respectively. Detection of precursor ions was set to 3 times at an absolute threshold of 5000 counts. The smart exclusion option was activated with exclusion after 15 acquired spectra and a release time of 0.5 min. MS-fragmentation analysis of the final fractions (anthocyanin A1, A2, A3, A4 and polyphenols) were conducted by directly injecting the previously with acetonitrile and 0.1% FA to a final anthocyanin concentration of about 0.1 µg/µL diluted samples into the ionization source of the UHR-TOF-MS instrument. Mass spectra were acquired and main molecular ions selected for MS-fragmentation analysis, at a flow rate of 100 µL/h, an accordingly adjusted collision energy specific for each compound, a width of 3 and a spectra rate of 1–3 Hz depending on the compound.

LC-UV/MS data were analyzed by means of Bruker Compass DataAnalysis version 4.1 software. The annotation of detected compounds is based on comparison of the measured retention times and molecular ion masses with available reference standards, as well as with literature data, and fragmentation pattern as obtained from LC-MS/MS experiments. 

### 4.4. Anthocyanin Isolation and Preparation of Blackberry AnthoKit

Based on 400 mL blackberry juice with an anthocyanin content of 0.25 µg/µL a juice raw extract was prepared, containing 100 mg of total anthocyanins for subsequent purifications. Therefore, the juice was first filtered through four layers of miracloth paper. Degreasing was realized by two-phase-partitioning, thoroughly mixing the extract with two volumes (*v*/*v*) of n-heptane and allowing phase separation overnight. The lower phase (degreased extract) has been carefully collected after phase separation. Rotary evaporation in vacuo was conducted to remove the remaining organic solvent. The extract was than adjusted to an end concentration of 2% methanol (MeOH) and 0.5% FA. A stepwise filtration of the so prepared defatted extract through nylon net filters was carried out down to 0.2 µm pore size (filtration in vacuum). This sample was named raw juice and was analyzed by LC-UV/MS and LC-MS/MS as stated above.

To obtain a preliminary separation of the various anthocyanins present in this mixture and to remove low molecular weight contaminations (e.g., salt and sugar), the raw juice was loaded on a solid phase extraction column (RediSep RF Gold^®^ C18Aq column, 15.5 g, 20–40 micron, 10–100 mg capacity, 13.5 mL column volume (CV), Teledyne Isco, Lincoln, NE, USA) equilibrated with 3 CV 2% methanol, 0.5% FA. Compound elution was realized by step gradients at 2%, 10%, 25%, 40% and 98% of MeOH with 0.5% FA using 7.4 CV each, at a flow rate of about 20 mL/min utilizing a Chromabond^®^ vacuum chamber (Machery-Nagel, Düren, Germany). Resulting fractions were analyzed by LC-UV/MS. Fractions containing anthocyanins were then used for further anthocyanin isolation, which were the three crude fractions from elution with 10, 25, and 40% MeOH, 0.5% FA. Other fractions containing polyphenols, but no anthocyanins, were combined and named polyphenols fraction. The volume and the content of organic solvent of these fractions were reduced under vacuum by means of rotary evaporation.

For final anthocyanin separation and purification, the isolated crude extracts were filtered through a cellulose acetate filter with 0.45 µm pore size, in vacuo. Subsequently the extracts were loaded on a preparative HPLC column (Gemini^®^ 5 µm NX-C18 110 A, LC Column 250 × 30 mm, AXIA™ Packed, Phenomenex, Aschaffenburg, Germany) equipped with an appropriate pre-column (SecurityGuard PREP Cartridge Gemini-NX C18 15 × 30 mm) and equilibrated with solvent A (water + 2% of 5% ammonium formiate in FA). Compound elution was realized by a linear gradient from 2–30% acetonitrile (solvent B) within 170 min at a flow rate of 10 mL/min utilizing a Varian ProStar chromatography instrument (Agilent Technologies, Santa Clara, CA, USA) with UV monitoring at 280 nm and 515 nm. Resulting fractions, showing anthocyanin specific absorption at 515 nm, were analyzed by LC-UV/MS and properly combined. Finally, four anthocyanin fractions enriched in the main blackberry anthocyanins Cy3glc (further named anthocyanin A1), cyanidin-3*O*-xyloside (Cy3xyl; anthocyanin A2), cyanidin-3*O*-(malonylglucoside) (Cy3malglc; anthocyanin A3) and cyanidin-3*O*-(6″-dioxalylglucoside) (Cy3dioxglc; anthocyanin A4), were obtained. Again, other fractions containing polyphenols, but no anthocyanins, were combined and added to the polyphenol fraction. The volume and the content of organic solvent of the final pure isolate fractions were reduced under vacuum by means of a rotation evaporator. 

A final purification and enrichment step was performed to remove remaining low molecular weight contaminations (e.g., salt and sugar). Each fraction (anthocyanin A1, A2, A3, A4 and polyphenols) was adjusted to a final concentration of 2% methanol, 0.5% FA. In addition, 10 mL of the original blackberry juice were mixed with 10 mL of 2% methanol, 0.5% FA and then cleared by filtration through a cellulose acetate filter with 0.45 µm pore size, in vacuum. Each fraction (A1, A2, A3, A4, polyphenols and the cleared blackberry raw juice) was loaded on a separate solid phase extraction cartridge (Chromabond^®^ C18, 70 mL, 10 g, Macherey-Nagel), which was conditioned with 3 CV of 2% methanol, 0.5% FA. The flow through was observed to be clear and the anthocyanins bound to the C18 column matrix. Remaining sugars and salts were washed off with two column volumes of 2% methanol, 0.5% FA. Elution of the bound anthocyanins and polyphenols was performed with 98% methanol, 0.5% FA. 

For preparation of the AnthoKit mix we combined the individual purified anthocyanin fractions based on their natural composition in the blackberry juice; namely 90% (*w*/*w*) of anthocyanin A1, 1% of anthocyanin A2, 2% of anthocyanin A3 and 7% of anthocyanin A4, as illustrated in Supplementary Figure S2. All resulting fractions (anthocyanin A1, A2, A3, A4 and ni. polyphenols) and the AnthoKit mix were dried under liquid nitrogen. The obtained dry powder was covered with argon and stored at −20 °C until further use.

### 4.5. Determination of Glucose, Fructose and Sucrose

Determination of soluble sugars in each fraction (anthocyanin A1–A4, polyphenols and the cleared blackberry raw juice) was performed as described previously through sequential enzymatic degradation of glucose, fructose and sucrose; using one unit each of glucose-6-phosphate dehydrogenase (Hoffmann-La Roche, Basel, Switzerland) for baseline generation, and then hexokinase (Sigma-Aldrich, Munich, Germany), phosphogluco-isomerase (Sigma-Aldrich) and invertase (β-fructofuranosidase, Sigma-Aldrich), respectively [26,55]. Quantities were calculated based on reference curves for authentic standards in the range from 0.1 mM to 1.0 mM.

### 4.6. Cell Culture

THP-1 monocytes were purchased from the German Collection of Microorganisms and Cell Cultures (DSMZ, Braunschweig, Germany). THP-1 cells were cultured in RPMI 1640 cell culture medium containing 2 g/L glucose (Thermo Fisher Scientific, Vienna, Austria) and supplemented with 10% heat inactivated fetal calf serum and 45 units/mL (1%) penicillin/streptomycin (Thermo Fisher Scientific) at 37 °C and 5% CO_2_ in a humidified atmosphere. The THP1-Lucia^TM^ NF-κB cells used for NF-κB activity measurements were purchased from Invivogen (Toulouse, France). THP1-Lucia NF-κB cells were maintained in RPMI 1640 cell culture medium containing 4.5 g/L glucose and 2.383 g/L HEPES (Thermo Fisher Scientific) supplemented with 10% (*v*/*v*) heat inactivated FCS, 1% penicillin/streptomycin and 100 µg/mL of normocin (Invivogen, Toulouse, France) at 37 °C and 5% CO_2_ in a humidified atmosphere. To maintain the stability of the reporter gene cell clone, cells were grown under selection pressure with 100 µg/mL of zeocin (Invivogen, Toulouse, France) every second cell passage. Cell density was kept between 2∙10^5^ and 1∙10^6^ cells/mL and the number of cell passage did not exceed 20 passages.

### 4.7. THP-1 Cell Differentiation

THP-1 and THP1-Lucia™ NF-κB monocytes were seeded into six-well plates at a cell density of 1.2∙10^5^ cells/mL and were subsequently differentiated into macrophages during 72 h with PMA (10 ng/mL) as described by Park et al. [56] and Kollarova et al. [27]. The cell culture medium for differentiation was removed and THP-1 macrophages were subsequently cultivated for 24 h in PMA-free culture medium prior the incubation with the test compounds. 

### 4.8. Cell Viability Assay

Cell viability after 2 h of pre-incubation with blackberry AnthoKit components (0.005–5 µg/mL), Cy3glc, Cy, PCA and PGA (0.005–10 µM) and subsequent additional stimulation with LPS from *E. coli* (10 ng/mL, 18 h) was tested by performing the alamar blue (Thermo Fisher Scientific) cell viability assay [32]. The non-fluorescent assay reagent resazurin is absorbed by living cells. In the cytosol it is reduced to resorufin which is highly fluorescent. For assay performance, the cell culture medium was removed from monocytes after the incubation with test compounds, cells were washed with PBS and incubated with resazurin in FCS-free cell culture medium at a final concentration of 10% (*v*/*v*). After 1 h of incubation at 37 °C and 5% CO_2_ an aliquot of the cell culture medium was transferred into a 96-well plate and the fluorescence of the reduced resazurin was measured with the Gen5 Microplate Reader (BioTek Instruments, Vienna, Austria) using a fluorescence excitation wavelength of 530 nm. The read out of the fluorescence emission was conducted at 560 nm.

### 4.9. Quantitative Real-Time PCR

Quantitative real-time PCR (qRT-PCR) was used for transcription analysis of IL-6, IL-8, TNF-α and IL-10 cytokines as well as miR-16, miR-125b, miR-155 and miR-146a. THP-1 derived macrophages were pre-incubated for 2 h with respective anthocyanins at concentrations between 0.0024–5 µg/mL or in the case of the degradation products Cy, PCA and PGA at concentrations of 0.005, 0.05, 0.5 and 10 µM with a final solvent concentration of 0.1% (*v*/*v*). Subsequently, cells were co-incubated with LPS (10 ng/mL) for 3 h for cytokine gene transcription analysis, or for 18 h for miRNA transcription measurements. The duration for LPS stimulation was chosen according to the monitoring of Chanput et al. showing that LPS-induced IL-6, IL-8 and TNF-α transcript levels reached their optimum between 3 and 6 h [33]. Dex (1 µM) and 0.1% of EtOH (80%) served as positive and solvent control, respectively. After cell lysis with Qiazol (Qiagen, Hilden, Germany) total RNA was extracted using the miRNeasy Kit (RNA size ≥ 18 nucleotides, Qiagen) according to the instructions of the manufacturer’s protocol. RNA purity and quantity was determined with the NanoDrop 2000 (Thermo Fisher Scientific). Following the instructions of the manufacturer’s protocol, 1 µg of total RNA was reverse transcribed into complementary DNA (cDNA) using the miScript II RT Kit (Qiagen). Gene specific cDNA was exponentially amplified by performing RT-PCR using the StepOne™Plus System (Applied Biosystems, Thermo Fisher Scientific). qRT-PCR was conducted in a 96-well plate using the miScript SYBR Green PCR Kit (Qiagen) and the following gene- or miRNA-specific primer assays (Qiagen). QuantiTect Primer Assays: Hs_GAPDH_1_SG; Hs_ACTB_1_SG; Hs_TNF_1_SG; Hs_IL6_1_SG; Hs_IL10_1_SG; RT^2^ qPCR Primer Assay: human IL8; miScript Primer Assays: Hs_RNU6-2_11 (detected transcript: NR_002752); Hs_SNORD68_11 (detected transcript: NR_002450); Hs_miR-16_2 (sequence: 5′UAGCAGCACGUAAAUAUUGGCG); Hs_miR-125b_1 (sequence: 5′UCCCUGAGACCCUAACUUGUGA); Hs_miR-146a_1 (sequence: 5′UGAGAACUGAAUUCCAUGGGUU); Hs_miR-155_2 (sequence: 5′UUAAUGCUAAUCGUGAUAGGGGU). The amplification protocol consisted of an initial activation step of the HotStarTaq polymerase for 15 min at 95 °C, 40 cycles of denaturation for 15 s at 94 °C, annealing for 30 s at 55 °C and extension for 30 s at 70 °C, followed by melting curve analysis. Relative gene transcript and miRNA transcript levels were calculated by applying the 2^−ΔΔCt^ method as described by Schmittgen and Livak [57,58]. Ct-values of the mRNA and miRNA target genes were normalized to the average of the Ct-values of the control genes ACTB and GAPDH or RNU6 and SNORD68, respectively. Subsequently, normalized data were compared to the calibrator (respective control sample).

### 4.10. Multiplex Immunoassay

In order to study the impact of selected polyphenols on TNF-α, IL-6 and IL-10 cytokine release a multiplex immunoassay was performed. For this, differentiated THP-1 macrophages were pre-incubated for 2 h with respective anthocyanins and their degradation products Cy, PCA and PGA (0.0024–5 µg/mL and 0.005, 0.05, 0.5 and 10 µM, respectively) and subsequently stimulated with LPS (10 ng/mL) for 18 h. The time of incubation with LPS was selected according to the results described by Chanput et al. [33], where THP-1 macrophages reached a plateau for TNF-α and IL-6 secretion after 18 h of incubation. Afterwards, supernatants were removed and centrifuged at 10.000× *g* (4 °C) to separate secreted cytokines from cell debris. Samples were stored at −80 °C until further analysis. To determine the cytokine concentration of TNF-α, IL-6 and IL-10, the supernatants were prepared with the ProcartaPlex Human Basic Kit and ProcartaPlex Human TNF-α, IL-6 and IL-10 Simplex (Affymetrix, Vienna, Austria) according to the instructions of the manufacturer’s protocol. Briefly, a mixture of magnetic beads was dispensed in a 96-well plate, carrying antibodies against the respective target cytokines, and 50 µL of each cytokine sample was added per well and incubated for 2 h. Once the cytokines attached to the corresponding antibody, a second cytokine dependent antibody was added and incubated for 30 min. The secondary antibody allowed afterwards the attachment of the reporter streptavidin-phycoerythrin conjugate (SA-PE), that permits the quantification of secreted cytokines with the Luminex 200 System (BioRad, Vienna, Austria), a bead-based flow cytometer, measuring the fluorescence of the bound SA-PE.

### 4.11. NF-κB Reporter Gene assay

The question, whether the NF-κB signaling pathway is affected by blackberry anthocyanins or the degradation product PGA was addressed by applying a NF-κB reporter gene assay. After differentiation into macrophages, NF-κB-inducible Luc reporter cells (THP1-Lucia™ NF-κB cells) were pre-incubated for 2 h with Cy3glc or PGA (0.005–10 µM) and subsequently stimulated with LPS (10 ng/mL) for 18 h, as recommended in the manufacturer’s protocol to induce luciferase gene expression by activation of NF-κB. The quantification of secreted Lucia luciferase in cell culture media is based on the catalytic oxidation of the coelenterazine-based luminescence assay reagent (QUANTI-Luc™) which leads to the emission of visible blue light (λ = 465–493 nm). For this, an aliquot of luciferase containing supernatant was transferred (10 µL) into a 96-well plate and fully automated 50 µL of QUANTI-Luc™ (Invivogen, Toulouse, France) were injected into each well with a dispenser. The resulting luminescence was measured with the Gen5 Microplate Reader (BioTek Instruments).

### 4.12. Statistical Analysis

Presented data are the means + SD of at least three independent biological replicates. The one sample Student’s *t*-test was used to compare significant differences between LPS-stimulated cells and non-stimulated cells, cells treated with the compound of interest or Dex treated cells (positive control), respectively. To verify significant differences between the test concentrations of the test-substance the analysis was performed by using one-way ANOVA followed by Holm-Bonferroni post-hoc test. The obtained results were considered as statistically different with *p* ≤ 0.05.

## 5. Conclusions

In the present study, the immunomodulatory properties of purified blackberry anthocyanins as well as complex mixtures were evaluated in plasma-relevant concentrations. Corroborated by a body of existing literature, Cy3glc showed anti-inflammatory properties on both transcription and protein level. Cy3dioxglc, on the contrary, showed potent pro-inflammatory properties as it not only induced pro-inflammatory cytokines but also significantly activated the NF-κB pathway. In the complex blackberry extract, the effects of the single anthocyanins seem to be well balanced as no immunomodulatory effects could be observed. Reports on anthocyanins are mostly focused on their anti-inflammatory properties. However, this study demonstrated that also pro-inflammatory effects can occur depending on the applied concentration and composition. These effects should be considered when preparing fortified food supplements intended for regular consumption.

## Data Availability

The majority of data is presented in the article and the Supplementary Materials. Unpresented data can be provided on request by the corresponding author.

## Supplementary Materials

The following are available online at https://www.mdpi.com/article/10.3390/ijms221910483/s1.
