# Supplementary material for: Immunomodulatory Properties of Blackberry Anthocyanins in THP-1 Derived Macrophages
Source: Int J Mol Sci. 2021 Sep 28;22(19):10483. doi: 10.3390/ijms221910483 (PMC8508689; doi:10.3390/ijms221910483)
Supplement: Supplementary file 1 [file ijms-22-10483-s001.zip › ijms-1392329-supplementary.pdf]

Figure S1

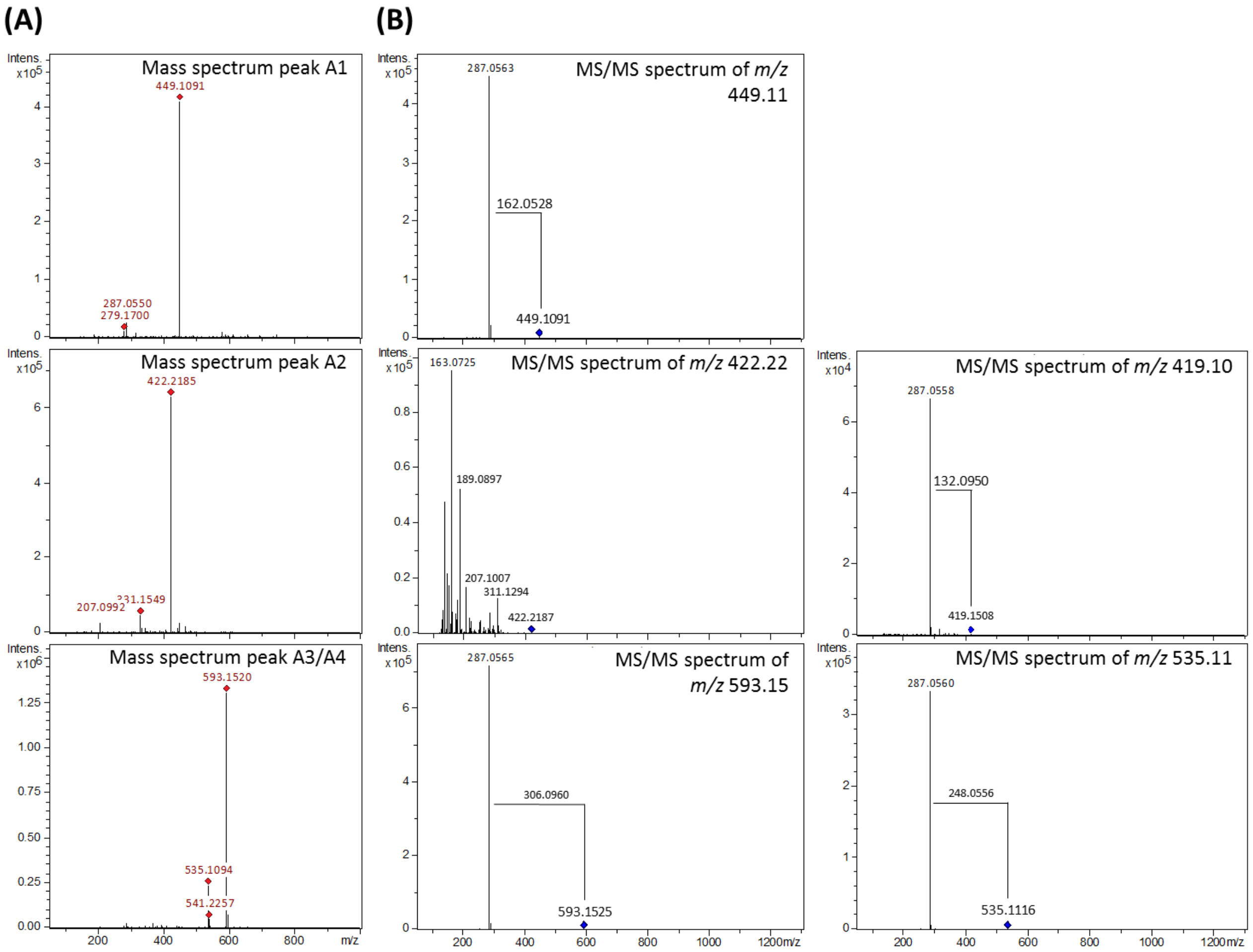

Supplementary Figure 1: Tentative annotation in blackberry *raw juice* based on MS/MS fragmentation pattern and comparison with literature data. (**A**) Sum of mass spectra from retention time 3.6-3.9 minutes (*anthocyanin A1*), 4.35-4.45 minutes (*anthocyanin A2* and *polyphenols PP*), and 4.9-5.0 minutes (*anthocyanin A3* and *anthocyanin A4*). (**B**) MS/MS fragmentation pattern of main molecular ions and annotation of released molecular features; n.a.: not annotated.

Figure S2

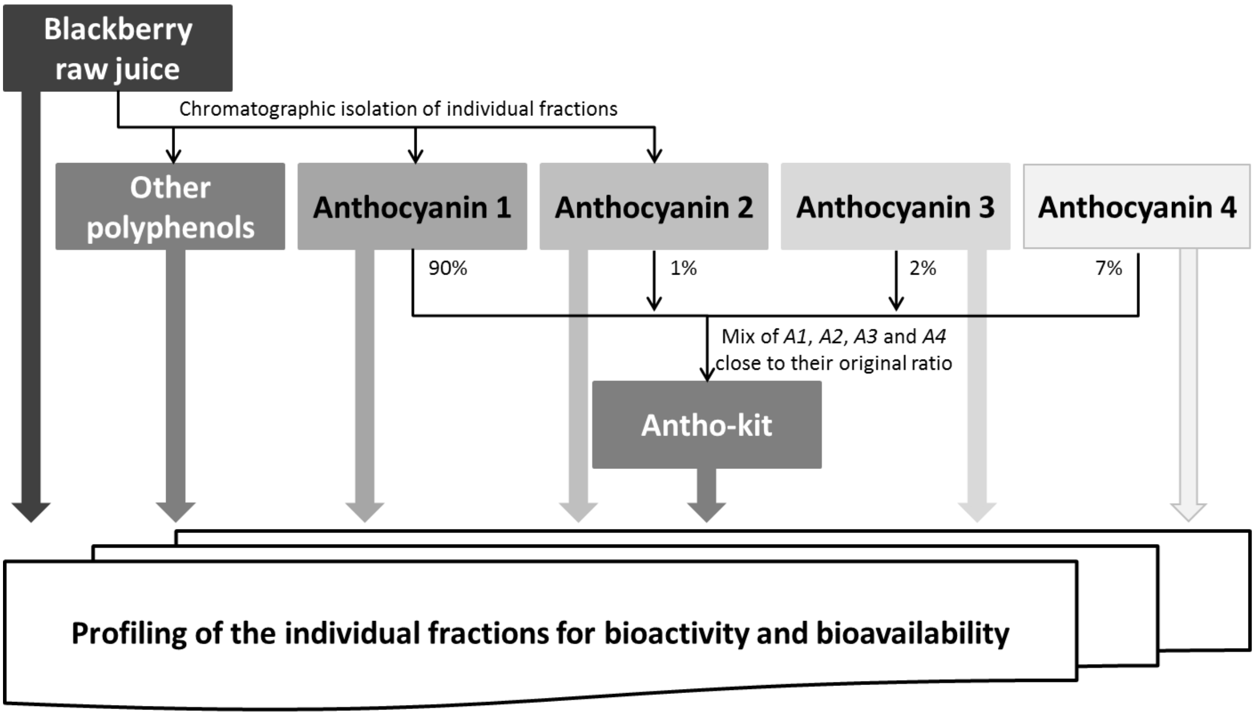

Supplementary Figure 2: Schematic presentation of the blackberry AnthoKit mix concept. From raw blackberry juice four individual anthocyanin fractions were isolated. Remaining material was collected as a fraction containing other polyphenols. Portions of the four anthocyanin fractions were mixed to an anthocyanin kit, based on their natural composition in the raw juice. All fractions (*raw juice*, *polyphenols*, *anthocyanin A1*, *A2*, *A3*, *A4*) and the mix were utilized in the bioactivity tests individually.

Figure S3

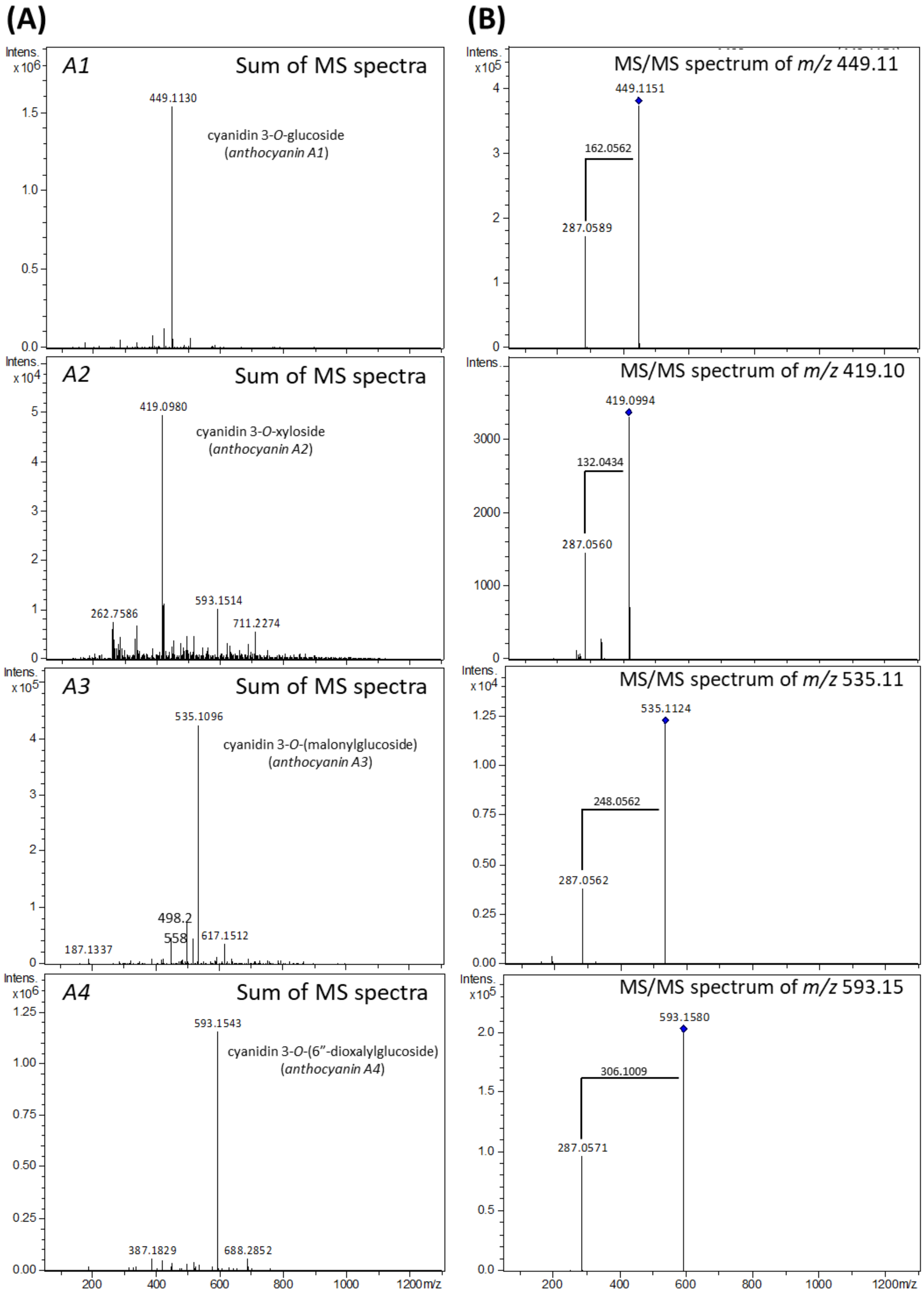

Supplementary Figure 3: Tentative annotation in isolated anthocyanin fractions based on MS/MS fragmentation pattern and comparison with literature data. (**A**) Sum of mass spectra of fraction *anthocyanin A1*, *A2*, *A3* and *A4*. (**B**) MS/MS fragmentation pattern of main molecular ions and annotation of released molecular features; n.a.: not annotated.

Figure S4

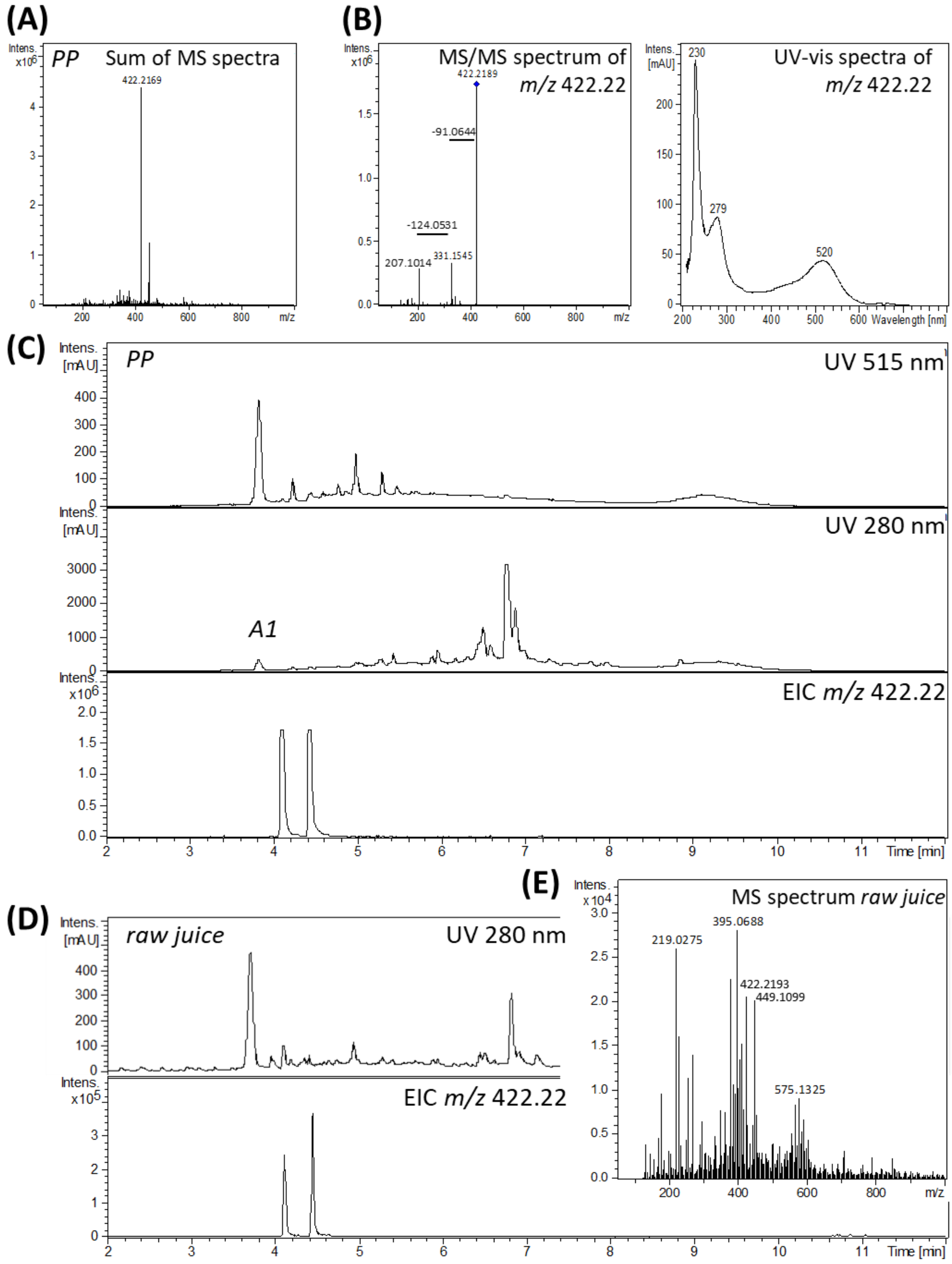

Supplementary Figure 4: LC-UV/MS analysis of blackberry *polyphenol* fraction (*PP*). (**A**) Sum of mass spectra acquired from MS direct injection, showing all detected masses and their abundances. (**B**) MS/MS fragmentation pattern of main molecular ion with *m/z* 422.22 and annotation of released

molecular features (left) and UV-vis spectra from 220-640 nm (right). (**C**) UV chromatogram at 515 nm (top) and 280 nm (middle); and extracted ion chromatogram (EIC) for *m/z* 422.22 generated for the *polyphenol* fraction resulted in two peaks at 4.10 and 4.43 minutes (bottom). (**D**) UV chromatogram at 280 nm and extracted ion chromatogram for *m/z* 422.22 generated for the *raw juice* fraction also resulted in two peaks at 4.10 and 4.43 minutes. (**E**) Sum of mass spectra acquired from MS direct injection of the *raw juice* fraction revealed *m/z* 422.22 as a major molecular ion.

Table S1: Anthocyanin and sugar concentration before and after chromatographic separation of blackberry juice. n.d. – not detectable

Fraction	Anthocyanin concentration [µg/µl]	Volume [ml]	Total anthocyanin amount [mg]	Sugar concentration [mM]		
				Glucose	Fructose	Sucrose
raw juice	0.25	400.00	100.00	6.61	9.16	0.65
anthocyanins A1	4.175	12.00	49.99	n.d.	0.01	n.d.
anthocyanins A2	0.17	6.00	1.03	0.01	0.01	n.d.
anthocyanins A3	0.22	6.00	1.34	0.02	0.01	0.01
anthocyanins A4	0.61	6.00	3.68	0.03	0.01	0.01
n.i. polyphenols	0.76	12.60	9.54	0.01	n.d.	n.d.

Figure S5

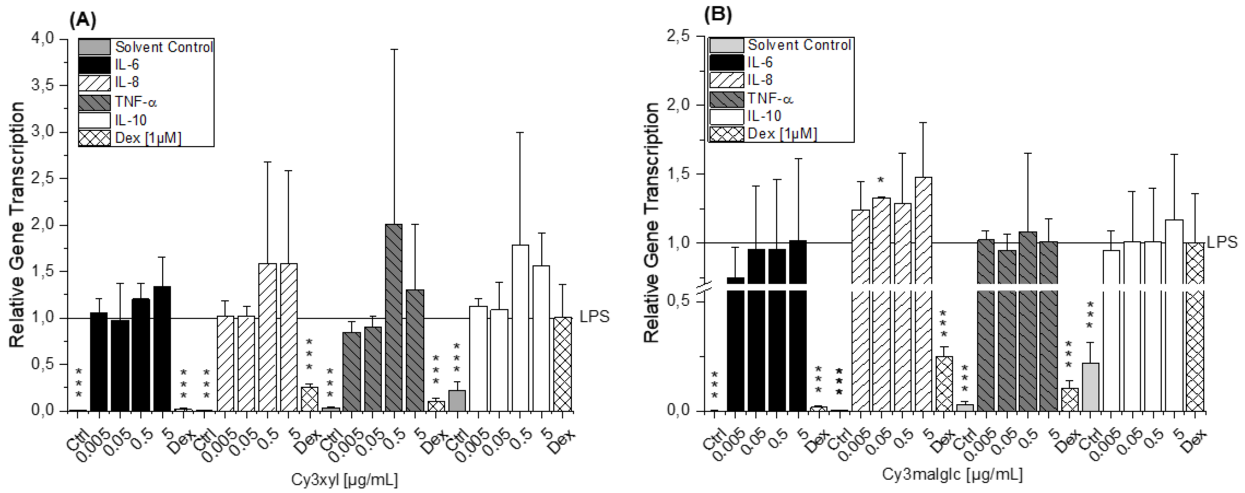

Supplementary Figure S5: Relative transcript levels of IL-6, IL-8 and TNF-α in THP-1 derived macrophages pre-incubated (2 h) with (**A**) Cy3xyl and (**B**) Cy3malglc and subsequently challenged by LPS for another 3 h. Dexamethasone (Dex; 1 µM) served as positive control. Cy3xyl = cyanidin-3*O*-xyloside; Cy3malglc = cyanidin-3*O*-(6”-malonylglucoside). Values shown are the means + SD of at least three independent experiments presented as relative gene transcription, normalized to β-Actin and GAPDH and compared to LPS stimulated cells (calibrator, y=1). Significant differences among the test concentrations were calculated by one-way ANOVA (p <0.05, a-c). Statistical differences compared to the LPS stimulus were calculated with a one-sample *t*-test (*p, **p, ***p <0.05, 0.01, 0.001).

Figure S6

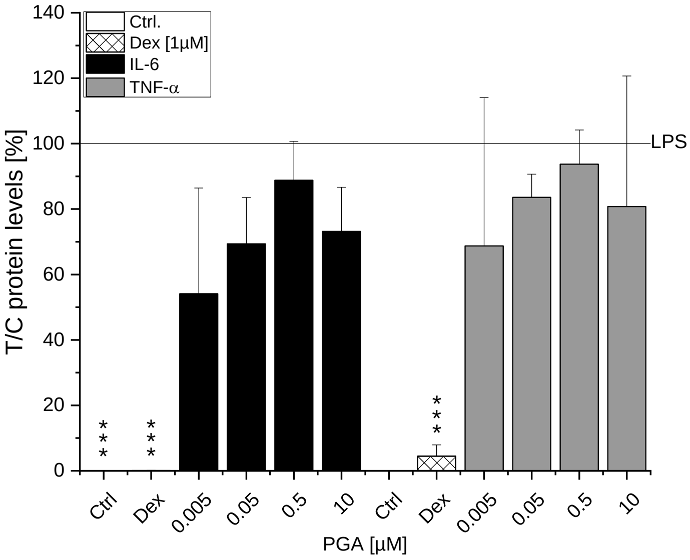

Supplementary Figure S6: Release of IL-6 and TNF-α from THP-1 derived macrophages pre-incubated (2 h) with PGA and subsequently additionally challenged by LPS for another 18 h. Dexamethasone (Dex; 1 µM) served as positive control. Values plotted are the mean + SD of three independent experiments and presented as test over control compared to LPS stimulated cells. Significant differences among the test concentrations were calculated with one-way ANOVA ($p < 0.05$, a-c). Statistical differences compared to the LPS stimulus were calculated with a one-sample t-test (*p, **p, ***p <0.05, 0.01, 0.001). No statistical evaluation could be performed for the TNF-α solvent control since measured levels equaled 0 ng/µL.

Figure S7

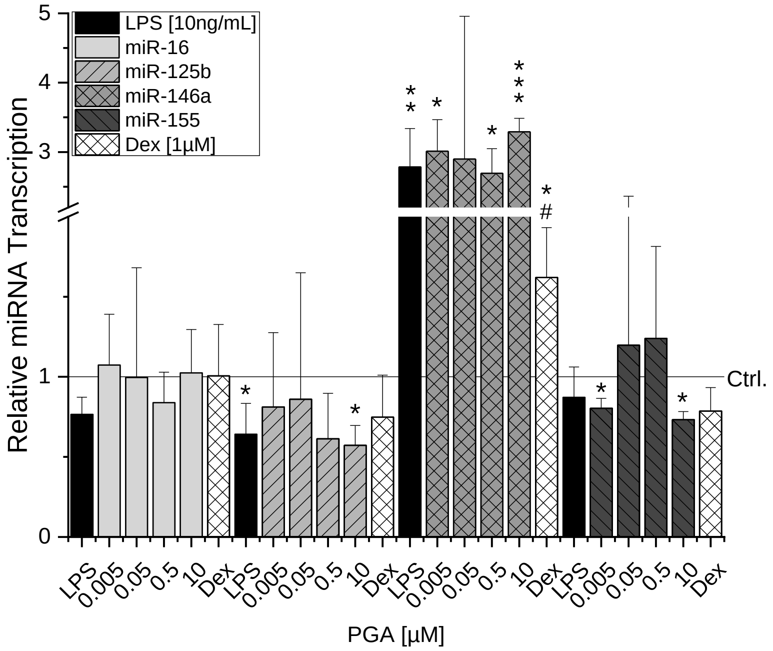

Supplementary Figure S7: miRNA levels of miR-16, miR-125b, miR-146a and miR-155 in THP-1 derived macrophages pre-incubated with PGA for 2 h and subsequently additionally challenged with LPS (10 ng/ml) for 18 h. Dexamethasone (Dex, 1 µM) served as a positive control. Values are the mean + SD of at least 3 independent experiments presented as relative gene transcription normalized to RNU6 and SNORD68 and compared to the solvent control (Ctrl., calibrator, y=1). Significant differences among the test concentrations were calculated by one-way ANOVA ($p < 0.05$, a-e). Statistical differences compared to the solvent control or the LPS stimulus were calculated with a one-sample *t*-test (*p, **p, ***p <0.05, 0.01, 0.001) or a two-sample *t*-test (#p <0.05), respectively.
